# *PROM1* and *PROM2* expression differentially modulates clinical prognosis of cancer: a multiomics analysis

**DOI:** 10.1038/s41417-019-0109-7

**Published:** 2019-06-05

**Authors:** Subbroto Kumar Saha, S. M. Riazul Islam, Kyung-Sup Kwak, Md. Shahedur Rahman, Ssang-Goo Cho

**Affiliations:** 1 Department of Stem Cell and Regenerative Biotechnology, Konkuk University, 120 Neungdong-ro, Gwangjin-gu, Seoul 05029 Republic of Korea; 2 Department of Computer Science and Engineering, Sejong University, 209, Neungdong-ro, Gwangjin-gu, Seoul 05006 Republic of Korea; 3 School of Information and Communication Engineering, Inha University, 100, Inha-ro, Nam-gu, Incheon 22212 Republic of Korea; 4 Department of Genetic Engineering and Biotechnology, Jashore University of Science and Technology, Jashore, 7408 Bangladesh

**Keywords:** Targeted therapies, Biomarkers, Cancer genomics

## Abstract

Prominin 1 (*PROM1*) is considered a biomarker for cancer stem cells, although its biological role is unclear. Prominin 2 (*PROM2*) has also been associated with certain cancers. However, the prognostic value of *PROM1* and *PROM2* in cancer is controversial. Here, we performed a systematic data analysis to examine whether prominins can function as prognostic markers in human cancers. The expression of prominins was assessed and their prognostic value in human cancers was determined using univariate and multivariate survival analyses, via various online platforms. We selected a group of prominent functional protein partners of prominins by protein-protein interaction analysis. Subsequently, we investigated the relationship between mutations and copy number alterations in prominin genes and various types of cancers. Furthermore, we identified genes that correlated with *PROM1* and *PROM2* in certain cancers, based on their levels of expression. Gene ontology and pathway analyses were performed to assess the effect of these correlated genes on various cancers. We observed that *PROM1* was frequently overexpressed in esophageal, liver, and ovarian cancers and its expression was negatively associated with prognosis, whereas *PROM2* overexpression was associated with poor overall survival in lung and ovarian cancers. Based on the varying characteristics of prominins, we conclude that *PROM1* and *PROM2* expression differentially modulates the clinical outcomes of cancers.

## Introduction

Cancer is one of the leading causes of human death and it has a profound impact on global health. According to the Surveillance, Epidemiology, and End Results Program’s (SEER) Cancer Statistics Review (CSR) 1975–2014, the number of new cases of cancer in the United States of America alone for all sites combined was 442.7 per 100,000 (both men and women) per year [1]. Cancer constantly introduces new challenges to various stakeholders, including researchers, medical practitioners, industrialists, and economists attempting to implement optimal health solutions.

Cumulative genetic alterations in the form of base insertions, deletions, substitutions, duplications, and translocations can be considered as the basis of cancer. Fortunately, targeted therapies or biological therapies based on the use of either molecular medicine or nano-engineered enzymes have been relatively successful in blocking the growth of cancer and cancer stem cells (CSCs) [2]. Owing to their self-renewing, multipotent, and high proliferative capacities, CSCs play a pivotal role in tumor invasion and metastasis. Therefore, targeting CSCs is critical for achieving high therapeutic efficiencies and preventing tumor recurrence. To develop targeted therapies, it is necessary to first identify suitable markers for identifying CSCs. A member of the prominin family, prominin 1 (*PROM1*), is considered a valuable marker of stem cells and CSCs [3–5]. Prominin family homologues in mammals consist of five transmembrane domains, an N-terminal domain exposed to the extracellular space, two small cytoplasmic loops, two large glycosylated extracellular loops, and a cytoplasmic C-terminal domain [6–9]. *PROM1* is commonly reported as a marker of neuronal and hematopoietic stem cells [4], but it is also expressed in CSCs and cancer cells, including in breast cancer [5], acute myeloid leukemia (AML) [10], and various pediatric brain tumors [11]. In addition, *PROM1* is involved in maintaining microvillar architecture and dynamics [12]. Recent studies suggest that *PROM1* is upregulated in non-small cell lung cancer tissue compared to normal lung tissue, and mutations in *PROM1* are associated with poor prognosis [13, 14]. High levels of *PROM1* mRNA are also associated with poor prognosis in pediatric medulloblastoma [15]. Moreover, *PROM1* regulates metastasis, drug resistance, and stemness properties in various cancer cells [3, 16, 17]. It can also regulate the activation of stem cells by orchestrating ciliary dynamics, and the absence of *PROM1* allows stem cells to resist the effects of sonic hedgehog (SHH) on growth stimulation, thereby disrupting stem cell activation [18]. Previous studies have shown that therapies targeting *PROM1* may prevent tumor development in various human cancers [19–22]. Although *PROM1* has been studied as a CSC marker and a regulator of cancer progression and prognosis over the last two decades, specific studies regarding the relationship between *PROM1* expression and prognosis in certain cancers are lacking.

*PROM2*, the second member of the prominin family, is structurally related to *PROM1*, but is encoded by a separate gene [6]. *PROM2* expression is limited to epithelial cells, where it may be involved in the organization of plasma membrane microdomains [6]. Furthermore, *PROM2* causes cell protrusions that recruit cholesterol with the aid of lipid rafts and subsequently, increase the phosphorylation of caveolin-1 in membrane microdomains [23]. A whole-genome expression profiling study reported that *PROM2* is overexpressed in endothelial cells in lung cancer [24]. Moreover, several other expression profiling studies have reported that *PROM2* is upregulated in various cancers including breast, brain, lung, renal, and tongue cancers and melanoma [6, 25, 26]. Despite this research, studies on *PROM2* expression in cancer and its relevance to clinical outcomes are still limited. Moreover, to the best of our knowledge, *PROM1* and *PROM2* genes have not yet been studied using data mining approaches. Therefore, this is the first systematic analysis of the possible role of *PROM1* and *PROM2* in various cancers, based on publicly available gene expression and clinical data.

Here, we aimed to identify the role of prominins in cancer progression and their value in cancer prognosis. As these proteins may exert their effects through signaling pathways, we hypothesized that a multiomics data mining approach could identify the link between prominin expression and clinical outcomes in cancer patients. Therefore, we investigated the patterns of expression, mutation, and copy number alteration of *PROM1* and *PROM2* genes to determine their clinical significance in human cancers through systematic data analysis. Moreover, we aimed to determine the combined prognostic significance of *PROM1* and *PROM2* in certain cancers using a multivariate prognosis analysis. We also analyzed the interacting partners and genes co-expressed with *PROM1* and *PROM2* in various cancers and subsequently, analyzed these genes to predict the probable underlying signaling pathways involved. These results provide useful information to facilitate the development of new approaches for anti-cancer therapies that target cancer stem cells.

## Methods

### mRNA expression analysis using Oncomine

Data regarding *PROM1* and *PROM2* mRNA expression in various cancer types was retrieved from the online database, Oncomine (https://www.oncomine.org/resource/login.html) [27, 28]. This database platform contains a large collection of independent datasets and expertly curated data. It can be used to identify novel targets for drug development and to interrogate gene expression profiles along with clear and consistent interpretation of results. Differences in mRNA expression between cancer tissues and their normal tissue counterparts were calculated using the following threshold parameters: *p* < 0.01, fold-change > 2, and gene ranking in the top 10%. The details of the analyses are summarized in Supplementary Tables 1 and 2. The co-expression profiles of prominins in different cancer types were also extracted from Oncomine and are illustrated as a heat map in Fig. 7.

### mRNA expression analysis using GEPIA

Gene expression profiling interactive analysis (GEPIA, http://gepia.cancer-pku.cn/) is a newly developed interactive online platform for analyzing RNA sequencing data [29]. It provides access to a large collection of data from 9736 tumors and 8587 normal samples from The Cancer Genome Atlas (TCGA) and the Genotype-Tissue Expression (GTEx) project. GEPIA was used for differential expression analysis of *PROM1* and *PROM2* in tumor/normal tissue from various cancers. GEPIA also provides other customizable functions, including patient survival and correlation analyses.

### Survival analysis using Kaplan–Meier plotter

The web-based tool, Kaplan-Meier plotter, was used to analyze the impact of 54,675 genes on survival using HGU133 Plus 2.0 array data from 10,461 cancer samples. Of these samples, 5143 were from breast cancer patients, 1816 from ovarian cancer patients, 2437 from lung cancer patients, and 1,065 from gastric cancer patients, who underwent an average follow-up period of 69, 40, 49, and 33 months, respectively. The main objective of this tool is to perform a meta-analysis-based biomarker assessment. In this study, the correlations between prominin expression and patient survival were analyzed using the Kaplan–Meier plotter (http://kmplot.com/analysis/) [30]. According to various quantiles of biomarker expression, the tool divides patient samples into pairs of groups to analyze the prognostic value of a particular gene. Kaplan–Meier survival plots were constructed to compare the two patient groups and calculate the log-rank *p*-value and the hazard ratio, with 95% confidence intervals.

### Prognosis analysis using PrognoScan

PrognoScan (http://dna00.bio.kyutech.ac.jp/PrognoScan/) [31] is a database that is used for the meta-analysis of the prognostic value of various genes. This online platform assists in investigating the relationship between gene expression and patient prognosis across a large collection of cancer microarray datasets. The correlation between prominin expression and survival was investigated in several cancer types using this tool. The significance threshold was adjusted to a Cox *p*-value < 0.05. These results are briefly presented in Supplementary Tables 3 and 4.

### Survival analysis using OncoLnc

OncoLnc (http://www.oncolnc.org/) is a web-based interactive tool for analyzing survival correlations and retrieving clinical data matched with expression data for mRNAs, miRNAs, and long non-coding RNAs [32]. It is a large collection of clinical data from 8,647 patients across 21 cancer studies from the TCGA and MiTranscriptome beta collections. Using this platform, Cox regression analysis data were acquired for *PROM1* and *PROM2* in up to 21 cancers. These data were then used to generate Kaplan-Meier plots for further analysis of RNAs of interest.

### Survival analysis using SurvExpress

SurvExpress (http://bioinformatica.mty.itesm.mx/SurvExpress) is a cancer-wide gene expression database with clinical outcomes and a web-based tool for survival analysis [33]. This database contains more than 39,000 samples and 225 datasets covering tumors in more than 26 different tissues. Using this platform, survival plots were generated for specific cancer types, using TCGA and GEO microarray data. Biomarkers can be assessed in several ways using this tool. For example, specific genes can be switched on and off, samples can be framed by available clinical information, and training and test samples can be identified.

### Correlation and survival analysis using the R2 platform

R2 (http://r2platform.com) is a genomics analysis and visualization platform with a database, coupled to a web-interface that provides a set of analysis tools [34]. R2 supports all types of survival data (e.g., overall survival) and can also be used to generate a Kaplan-Meier plot for a specific dataset. The Kaplan Scan feature was used to establish the optimum cut-off values for *PROM1* and *PROM2*, based on the *p*-value from a log-rank test of the cancers of interest. Although the Kaplan Scan tool was applied, the binary heat map was also used to show clustering based on “good” vs. “bad” prognoses. The R2 software was also used to identify genes that correlated with *PROM1* and *PROM2*, using the freely available TCGA datasets on the R2 website. To import the list of common genes that correlated with *PROM1* or *PROM2* for all tumors of interest, a Venn diagram was generated using the tool, Venny 2.1 [35]. To understand how these common correlated genes collectively regulate signaling pathways, gene ontology and pathway analyses were performed using the Protein Analysis Through Evolutionary Relationships (PANTHER) tool (http://pantherdb.org/) [36]. This is an online system that classifies proteins (and their genes) in terms of various ontologies, including molecular function, biological process, cellular components, and pathway.

### Analysis of gene expression and mutations using cBioPortal

An integrative analysis of *PROM1* and *PROM2* and clinical characteristics was performed using cBioPortal for Cancer Genomics (http://www.cbioportal.org), which is an open-access and open-source resource for the interactive visualization and analysis of multidimensional cancer genomics data sets [37, 38]. At the time of this study, 56,250 tumor samples from 215 cancer studies were available online. It contains various types of data, including DNA copy number, mRNA expression, non-synonymous mutations, DNA methylation, and limited de-identified clinical data. The query interface combined with personalized data storage enables interactive investigations of genetic alterations within specific genes, across available samples. The primary search parameters included alterations (amplifications, deep deletions, and missense mutations), copy number alterations (CNAs) from GISTIC, and RNA sequencing data, using the default settings. For the secondary search, we focused on RNA sequencing data.

### Protein-protein interaction (PPI) analysis using GeneMANIA

GeneMANIA (https://genemania.org/) is an online tool that provides information on hypothetical gene function, interrogates gene lists, and ranks genes based on functional evaluations [39]. It contains a large set of functional association data, including protein and gene interactions, pathways, and co-expression data. We applied the GeneMANIA analysis tool to predict PPIs using *PROM1* and *PROM2* as queries. The prediction output is in the form of a network that shows the relationships between genes in the list, where nodes symbolize genes and links represent networks.

### Statistical analysis

Bar and forest plots were drawn using GraphPad Prism version 7 (GraphPad Software, La Jolla, CA, USA). Survival curves were constructed using PrognoScan, PROGgeneV2, OncoLnc, and Kaplan-Meier plotters. All results are displayed with *p*-values obtained from a log-rank test. The levels of significance (*p*-values) of the Oncomine and heat map data were determined by the programs used for the analyses. An unpaired *t*-test was performed to analyze two groups (normal vs. cancer). For multivariate survival analysis, clinical outcome data were retrieved from the TCGA database using OncoLnc. The data were then processed to generate survival curves using GraphPad Prism version 7 software. Cox regression analysis was performed for univariate and multivariate survival analysis to define the independent factors that had a significant effect on patient survival. Log-rank *p*-values < 0.05 were considered significant and *p* = ns denoted results that were not significant.

## Results

**Fig. 1:**
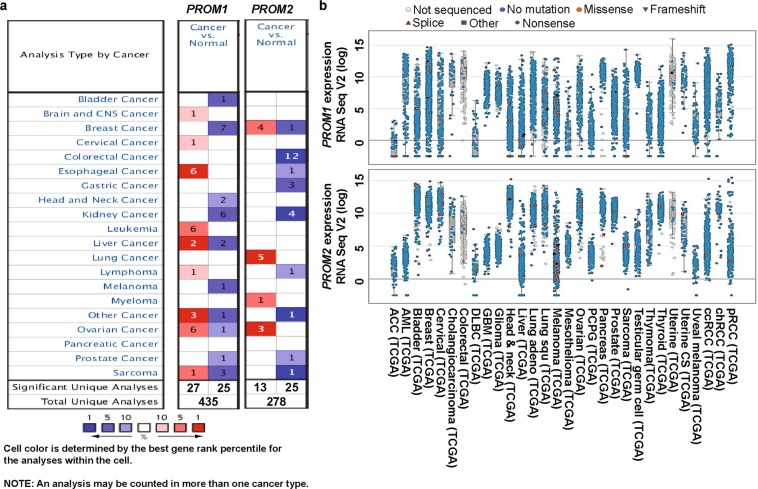
Transcription levels of PROM1 and PROM2 in different types of cancers (Oncomine and TCGA databases). **a** This graphic was generated using Oncomine, indicating the numbers of datasets with statistically significant (*p* < 0.01) overexpression (red) or underexpression (blue) of PROM1 and PROM2 mRNA (cancer vs. corresponding normal tissue). The threshold was designed with the following parameters: *p*-value = 0.01, fold change = 2, and gene ranking = 10%. The numbers in the boxes represent the number of analyses that met these thresholds. **b** Analysis of PROM1 and PROM2 mRNA levels in 30 types of human cancer using data from cBioPortal (http://www.cbioportal.org/index.do). Every dot represents a single study. White dots represent those without gene sequencing data, blue dots represent normal gene sequencing results (no mutation), and orange spots represent missense mutations. Flowchart: merged spots represent frame shifts, isosceles triangles represent splice sites, diamonds represent nonsense mutations, and rectangles represent others. The median and interquartile ranges are shown in each box

To understand the role of prominins in cancer, we compared their transcription levels in cancer tissues and normal tissues using the visualization tools provided by the Oncomine database. The various underlying threshold parameters, without altering any filter settings, were as follows: *p*-value, 0.01; fold change, 2; and gene ranking, 10%. Using these settings, we observed that prominins were overexpressed in some cancer tissues when compared to their expression levels in normal tissues and were underexpressed in others. These results indicate that the prominins may possess either oncogenic or anti-oncogenic characteristics, depending on the type of cancer (Fig. 1a). TCGA contains a large collection of RNA sequencing data and is useful resource for understanding the molecular basis of cancer. By accessing TCGA data via cBioPortal, we also further analyzed the mRNA levels of prominins in various types of cancer. *PROM1* and *PROM2* were found to be differentially expressed in many cancer types (Fig. 1b). The analyses of prominins are described in more detail below.

### Prominin mRNA expression analysis

**Fig. 2:**
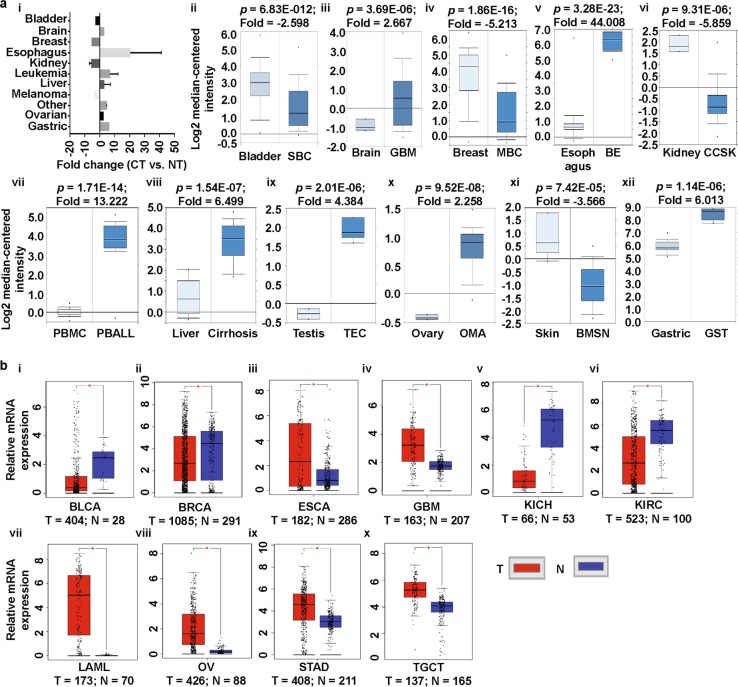
PROM1 expression analysis in different cancer types (Oncomine and TCGA databases). **a** The box plot comparing PROM1 expression in normal (left plot) and cancer tissues (right plot) were derived from the Oncomine database. The fold change of PROM1 in various cancer types was determined from the analyses shown in Supplementary Table 1 (**i**). Analysis of SBC relative to normal bladder (**ii**), GBM relative to normal brain (**iii**), MBC relative to normal breast (**iv**), BE relative to normal esophagus (**v**), CCSK relative to normal kidney (**vi**), PBALL relative to PBMC (**vii**), cirrhosis relative to normal liver (**viii**), TEC relative to normal testis (**ix**), OMA relative to normal ovary (**x**), BMSN relative to normal skin (**xi**) and GST relative to normal stomach (**xii**). The threshold was designed with the following parameters; *p*-value = 0.01, fold change = 2, and gene rank = 10%. **b** PROM1 expression in The Cancer Genome Atlas (TCGA) database. Box plots showing PROM1 mRNA expression in various tumor (T) and corresponding normal (N) tissues, using TCGA data from GEPIA (**i-x**). The threshold was designed with the following parameters: *p*-value = 0.01, fold change = 2. *SBC* superficial bladder cancer, *MBC* mucinous breast carcinoma, *BE* Barrett’s esophagus, *EA* esophageal adenocarcinoma, *CCSK* clear cell sarcoma of the kidney, *CRCC* chromophobe renal cell carcinoma, *PBMC* peripheral blood mononuclear cell, *PBALL* pro-B acute lymphoblastic leukemia, *TEC* testicular embryonal carcinoma, *OMA* ovarian mucinous adenocarcinoma, *OCCA* ovarian clear cell adenocarcinoma, *BMSN* benign melanocytic skin nevus, *GST* gastrointestinal stromal tumor, *BLCA* bladder urothelial carcinoma, *BRCA* invasive breast carcinoma, *CHOL* cholangiocarcinoma, *COAD* colon adenocarcinoma, *ESCA* esophageal carcinoma, *GBM* glioblastoma multiforme, *HNSC* head and neck squamous cell carcinoma, *KICH* kidney chromophobe, *KIRC* kidney renal clear cell carcinoma, *LAML* acute myeloid leukemia, *OV* ovarian serous cystadenocarcinoma, *PAAD* pancreatic adenocarcinoma, *READ* rectum adenocarcinoma, *STAD* stomach adenocarcinoma, *TGCT* testicular germ cell tumor, *THCA* thyroid carcinoma, *UCEC* uterine corpus endometrial carcinoma, *UCS* uterine carcinosarcoma

**Fig. 3:**
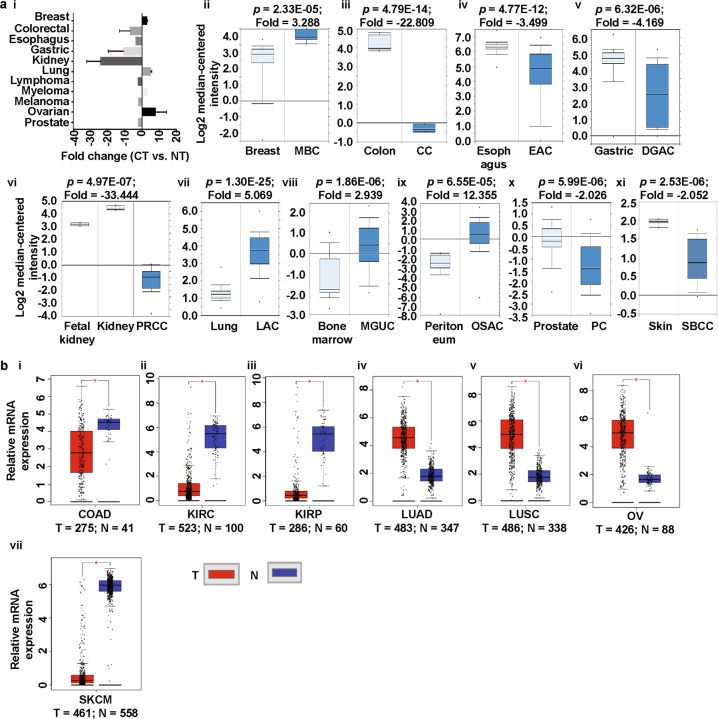
PROM2 expression analysis in different cancer types (Oncomine and TCGA databases). **a** The box plot comparing PROM2 expression in normal (left plot) and cancer tissues (right plot) was derived from the Oncomine database. The fold change of PROM2 in various cancer types was identified from our analyses presented in Supplementary Table 2 (**i**). Analysis of MBC relative to normal breast (**ii**), CC relative to normal colon (**iii**), EAC relative to normal esophagus (**iv**), DGAC relative to normal stomach (**v**), PRCC relative to normal kidney (**vi**), LAC relative to normal lung (**vii**), MGUC relative to normal bone marrow (**viii**), OSAC relative to normal peritoneum (**ix**), PC relative to normal prostate (**x**) and SBCC relative to normal skin (**xi**). The threshold was designed with the following parameters: *p*-value = 0.01, fold change = 2, and gene rank = 10%. **b** PROM2 expression data from the Cancer Genome Atlas (TCGA) database. Box plots showing PROM2 mRNA expression in various tumors (T) and corresponding normal (N) tissues using TCGA data from GEPIA (**i-vii**). The threshold was designed with the following parameters: *p*-value = 0.01, fold change = 2. *MBC* mucinous breast carcinoma, *CC* colon carcinoma, *EAC* esophageal adenocarcinoma, *DGAC* diffuse gastric adenocarcinoma, *PRCC* papillary renal cell carcinoma, *LAC* lung adenocarcinoma, *MGUC* monoclonal gammopathy of undetermined significance, *OSAC* ovarian serous adenocarcinoma, *PC* prostate carcinoma, *SBCC* skin basal cell carcinoma, *CESC* cervical squamous cell carcinoma and endocervical adenocarcinoma, *CHOL* cholangiocarcinoma, *KIRC* kidney renal clear cell carcinoma, *KIRP* kidney renal papillary cell carcinoma, *LUAD* lung adenocarcinoma, *LUSC* lung squamous cell carcinoma, *OV* ovarian serous cystadenocarcinoma, *PAAD* pancreatic adenocarcinoma, *SARC* sarcoma, *SKCM* skin cutaneous melanoma, *THYM* thymoma, *UCEC* uterine corpus endometrial carcinoma

To explore the expression patterns of prominins in various cancer types, we analyzed cDNA microarray data, using the differential analysis tool of the Oncomine database. The database was queried for prominin expression in each cancer type and in respective normal tissue, individually. The analysis showed that PROM1 was overexpressed in pro-B acute lymphoblastic leukemia, and brain, esophageal, liver, testis, ovarian, and gastric cancers, but underexpressed in bladder, breast, kidney, and skin cancers, compared to that in normal tissue (Fig. 2a [i-xii)], Supplementary Fig. S1, Supplementary Table 1) [40–49]. To confirm the PROM1 expression results obtained from the Oncomine database, we performed a single-gene analysis of PROM1 using another online platform, GEPIA. These results, as shown in Fig. 2b (i-x), confirmed PROM1 overexpression in brain, esophageal, leukemia, testis, ovarian and stomach cancers and PROM1 underexpression in bladder, breast, and kidney cancers. The pattern of PROM2 expression in different types of cancers was considerably different to the expression pattern of PROM1. We observed that compared to that in normal tissues, PROM2 was significantly overexpressed in breast, lung, bone marrow, and ovarian cancers, whereas it was underexpressed in colon, esophageal, gastric, kidney, prostate and skin cancers (Fig. 3a [i-xi], Supplementary Fig. S3, Supplementary Table 2) [42, 49–55]. The above expression pattern of PROM2 in colon, lung, ovarian, kidney, and skin cancers has also been reported from TCGA data on the GEPIA website (Fig. 3b [i-vii]). Note that TCGA-based GEPIA results on prominin mRNA expression are mainly used for validating the expression results obtained via Oncomine-assisted analysis. When we performed expression analysis using GEPIA, we also recorded the expression patterns of for some other cancers which are not available in Oncomine platform (Supplementary Fig. S2 and S4). The systematic analysis carried out here was able to assess the mRNA expression status of prominins across a wide range of cancer types. Since expression the prominin expression status was confirmed in multiple databases, these results can be considered reliable. These results regarding PROM1 and PROM2 expression are also supported by previous studies. For example, PROM1 overexpression has been shown in esophageal and ovarian cancers [56, 57]. Moreover, PROM1+ ovarian CSCs are highly tumorigenic, chemo-resistant, and metastatic and they promote the adhesion capabilities of the ovarian cancer metastatic niche [57]. In contrast, our analysis showed that PROM2 is overexpressed in breast and lung cancers, and this result is in agreement with those of previous genome profiling studies and studies on expressed sequence tag (EST) clones deposited in the GenBank database [58, 59]. Thus, these results imply that prominins may be either oncogenes or tumor suppressor genes, depending on the type of cancer. These results showing the dysregulation of prominins can potentially be translated into clinical practice. For example, PROM1 may be a biomarker for the prediction of lung metastasis and poor prognosis in patients with osteosarcoma, in which the expression of this gene is considerably high [60, 61]. PROM1 expression may also be a predictor of poor clinical outcome in patients with ovarian cancer [62]. Therefore, we next investigated the extent to which prominin expression is associated with prognosis.

### Estimation of the prognostic value of prominins

**Fig. 4:**
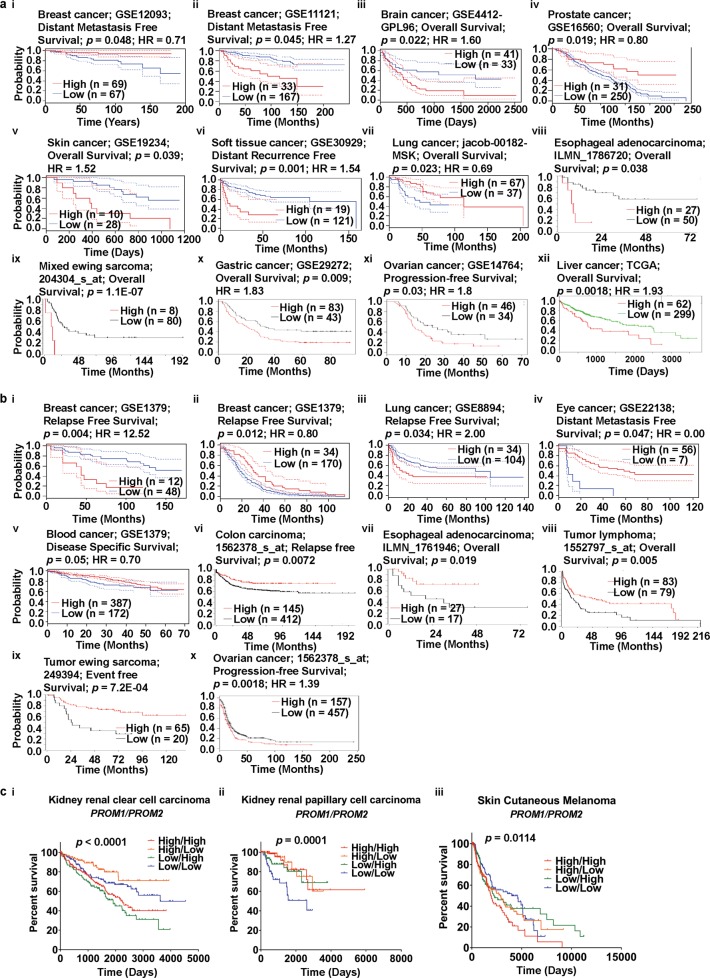
Correlation of PROM1 and PROM2 expression with prognosis of various cancers (PrognoScan, R2: Kaplan Meier Scanner, Kaplan–Meier plotter, SurvExpress, and OncoLnc). **a** Survival curves comparing patients with high (red) and low (blue) PROM1 expression were plotted using breast (**i** and **ii**), brain (**iii**), prostate (**iv**), skin (**v**), soft tissue (**vi**), and lung (**vii**) cancer data from PrognoScan; esophageal cancer (**viii**) and sarcoma (**ix**) data from R2: Kaplan Meier Scanner; gastric (**x**) and ovarian (**xi**) cancer data from Kaplan-Meier Plotter; and liver cancer (**xii**) data from SurvExpress. Cox *p*-value threshold < 0.05. **b** Survival curves comparing patients with high (red) and low (blue) PROM2 expression were plotted using breast (**i** and **ii**), lung (**iii**), eye (**iv**), and blood (**v**) cancer data from PrognoScan; colon cancer (**vi**), esophageal cancer (**vii**), lymphoma (**viii**), and sarcoma (**ix**) data from R2: Kaplan Meier Scanner; and ovarian cancer (**x**) data from Kaplan-Meier plotter. Cox *p*-value threshold < 0.05. **c** Co-expression of PROM1 and PROM2 with respect to the cancer patient prognosis. Multivariate survival curves comparing the prognosis of patients with high/high (red), high/low (orange), low/high (green), and low/low (blue) expression co-expression patterns of PROM1/PROM2 in KIRC (**i**), KIRP (**ii**), and SKCM (**iii**). Clinical outcome data were retrieved from TCGA using OncoLnc. *p* < 0.05 represents statistical significance. Abbreviations: KIRC - kidney renal clear cell carcinoma; KIRP - kidney renal papillary cell carcinoma; SKCM - skin cutaneous melanoma

To investigate the relationship between prominin gene expression and clinical prognosis, we used several online tools, namely, PrognoScan, R2, Kaplan-Meier plotter, SurvExpress, and OncoLnc. A positive correlation was observed between PROM1 overexpression and poor prognosis in brain, skin, and soft tissue cancers (Fig. 4a [iii, v, vi], Supplementary Fig. S5 [b–d], Supplementary Table 3), as analyzed using the PrognoScan database. In addition, low levels of PROM1 expression were correlated with poor overall survival (OS) in prostate and lung cancers (Fig. 4a [iv, vii], Supplementary Table 3). Therefore, these results indicated that PROM1 can be considered an oncogene for brain, skin, and soft tissue cancers, but a tumor suppressor gene for prostate and lung cancers. However, the relationship between PROM1 expression and survival in breast cancer was not clear, because of contradictory results (Fig. 4a [i, ii], Supplementary Fig. S5a, Supplementary Table 3). For example, an analysis of the GEO dataset, GSE12093, showed that the probability of long-term survival of a patient with breast cancer was higher with higher levels of PROM1 expression, whereas an analysis of dataset GSE11121, gave the opposite result. In addition, we found that high PROM1 expression was associated with poor OS in patients with esophageal cancers and mixed Ewing sarcoma (Fig. 4a [viii and ix]), whereas Ewing sarcoma patients with high PROM1 expression showed good prognosis (Supplementary Fig. S5e), as analyzed using the R2 platform. Similarly, in gastric, liver, and ovarian cancer patients, high levels of PROM1 expression were associated with poor OS (Fig. 4a [x-xii]). In kidney cancers, we observed a positive correlation between PROM1 expression and high rates of survival (Supplementary Fig. S5g). In liver cancer, mixed results were observed. Data from the SurvExpress database showed that PROM1 expression correlated positively with OS, whereas the Kaplan-Meier plotter showed that low PROM1 expression was associated with increased OS rates (Fig. 4a [xii]; Supplementary Fig. S5f). Interestingly, the Oncomine database also showed low and high expression of PROM1 in liver cancer and sarcoma (Fig. 1a) depending on the analysis. The source of the contradiction in survival results between Kaplan-Meier plotter and SurvExpress may be the inadequate number of studies and reports. Therefore, survival data for patients with liver cancer may be more precise if the merged data from the SurvExpress database and the Kaplan-Meier plotter database were analyzed.

Analysis of data from the PrognoScan database showed a significant correlation between PROM2 overexpression and poor relapse-free survival in patients with lung cancer (Fig. 4b [iii], Supplementary Table 4). In addition, low PROM2 expression was associated with poor prognosis in eye and blood cancers (Fig. 4b [iv and v], Supplementary Table 4). The relationship between PROM2 expression and survival in breast cancer patients is also questionable because of contradictory results (Fig. 4b [i and ii], Supplementary Fig. S6a and b, Supplementary Table 4). In this regard, the analysis of data from the PrognoScan database is in agreement with the results of Kaplan-Meier plotter data analysis. According to the latter, the association of PROM2 expression with survival of breast cancer patients is not straightforward and shows some inconsistencies (Supplementary Figs. S6c–e), depending on the receptor status of breast cancer cells. For instance, low PROM2 expression was associated with poorer relapse-free survival in HER2+ breast cancer patients compared to ER+ and PR+ breast cancer patients (Supplementary Figs S6f-h). We also found that HER2+ breast cancers showed an association between low survival rates and low PROM2 expression, whereas breast cancers with mutated p53 showed an association between low survival rates and high PROM2 expression (Supplementary Fig. S6h and i). Therefore, PROM2 expression is not a reliable prognostic marker of OS in patients with breast cancer. We also observed that both low and high levels of PROM2 expression were associated with poor survival in ovarian cancer patients (Fig. 4b [x]). In contrast, low PROM2 expression was associated with poor clinical outcomes in patients with colon and esophageal cancer, lymphoma, and sarcoma, using the R2 platform (Fig. 4b [vi-ix]).

The prognostic value of PROM1 and PROM2 expression levels for different cancer patients was also determined based on data from the PrognoScan database (Supplementary Tables 3 and 4). The poor prognosis seen in lung cancer patients with higher PROM2 expression (Fig. 4b [iii]) was in agreement with the analysis of Kaplan–Meier plotter data (Supplementary Fig. S6j). While high PROM1 expression was common in esophageal and liver cancers, based on data from Oncomine, R2, and SurvExpress (see Figs. 1a, 4a [viii and xii]), this gene was underexpressed in kidney cancer, according to the analysis of data from the Oncomine and OncoLnc databases (Fig. 1a and Supplementary Fig. S5g). In contrast, data from both the Kaplan-Meier plotter and PrognoScan databases confirmed high PROM2 expression in lung cancer (Figs. 1a, 4b [iii] and Supplementary Fig. S6j). In summary, a comprehensive analysis of survival data from a range of online resources, highlighted the oncogenic role of PROM1 in brain and ovarian cancers. However, the role PROM1 in liver and breast cancers was not clear. In contrast, the oncogenic role of PROM2 in lung cancer was obvious, unlike in breast cancer.

To investigate the relationship between prognosis and co-expression of PROM1 and PROM2, we retrieved clinical prognosis data from patients with various types of cancers, including breast, kidney, brain, ovarian, lung, and skin cancers, using OncoLnc, which accesses data from TCGA (Fig. 4c and Supplementary Fig. S7). The clinical prognosis data were then used to prepare a multivariate survival plot to assess the effect of high/high, high/low, low/high, and low/low expression of PROM1 and PROM2 in each cancer. The primary endpoint for this analysis was OS. The expression levels of both PROM1 and PROM2 were higher in ovarian cancer tissues than in their corresponding normal tissues, leading to poor prognosis (Fig. 4a [xi] and 4b [x]). Based on this expression pattern, we performed a multivariate survival analysis of PROM1/PROM2 co-expression in ovarian cancer. We did not observe a significant effect on survival probability among the high/high, high/low, low/high, and low/low groups (Supplementary Fig. S7). A similar result was also observed in several other cancers, including breast, brain, and lung cancers (Supplementary Fig. S7). The multivariate survival analysis revealed a significant association between low/high expression of PROM1/PROM2 and poorer prognosis of patients with kidney renal clear cell carcinoma (KIRC) compared to patients with high/high, low/low, or high/low expression patterns (Fig. 4c [i]). This result suggested that the partial-co-expression of PROM1 and PROM2 may regulate cancer prognosis. In the case of kidney renal papillary cell carcinoma (KIRP), the low/low expression pattern of PROM1/PROM2 was associated with poor prognosis compared to the high/high, high/low, and low/high patterns (see Fig. 4c [ii]). We next focused on skin cancer, where the high expression of both PROM1 and PROM2 was associated with poorer prognosis than the high/low, low/high, and low/low PROM1/PROM2 expression patterns (Fig. 4c [iii]). Thus, our multivariate survival analyses showed that the pattern of PROM1 and PROM2 co-expression modulated the clinical outcomes of patients with certain types of cancers, which may help our understanding of the underlying mechanism of cancer prognosis with respect to prominin expression. Furthermore, the interaction between these two prominins may be associated with the progression of various types of cancers.

### Predicting PPIs of prominins

**Fig. 5:**
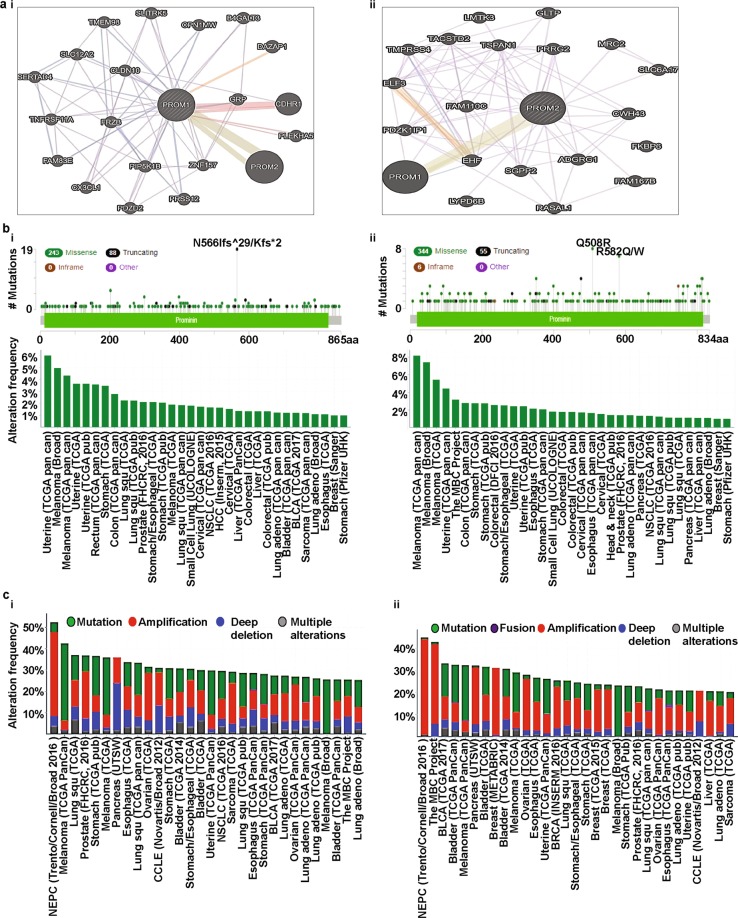
Identification of known and predicted structural proteins essential for PROM1 and PROM2 function (GeneMANIA) and frequency of mutations and copy number alterations (CNAs) in various types of cancer (cBioPortal web). **a** Interacting nodes are displayed in circles using GeneMANIA. Predicted functional partners of PROM1 (**i**) and PROM2 (**ii**) are shown after considering co-expression, co-localization, genetic interactions, pathways, physical interactions, and predicted shared protein domains. **b** In total, 331 mutation sites were identified and were located between amino acids 0 and 865 of PROM1. PROM1 mutations mainly occurred in uterine cancer and existed in a hotspot in the prominin domain (**i**). In total, 405 mutation sites were detected and were located between amino acids 0 and 834 of PROM2. PROM2 mutations mainly occurred in melanoma and also existed in a hotspot in the prominin domain (**ii**). **c** The alteration frequency of a ten-gene signature (PROM1, PROM2, GRP, ZNF157, FRZB, CLDN10, PIP5K1B, CDHR1, CX3CL1, and PDZD2) was determined using cBioPortal. Only data sets containing >100 samples per cancer type and an alteration frequency of >25% are shown. The alterations included mutations (green), amplifications (red), deep deletions (blue), or multiple alterations (grey) (**i**). The alteration frequency of a ten-gene signature (PROM2, PROM1, EHF, FAM110C, SGPP2, ADGRG1, CWH43, PRRG2, TSPAN1, and ELF3) was determined using cBioPortal. Only data sets containing >100 samples per cancer type and an alteration frequency of >20% are shown. The alteration frequency included mutations (green), fusions (brown), amplifications (red), deep deletions (blue), or multiple alterations (grey) (**ii**)

Studies have shown that PPIs are crucial events in cellular mechanisms that process downstream signaling and subsequently, affect cellular processes, including cell growth and division [63–65]. Accumulating evidence suggests that several factors, including DNA hypomethylation and hypoxia, affect PROM1 expression in cancer cells [66, 67]. However, unlike PROM1, the factors that affect PROM2 expression are still mostly unknown. As reported previously [68], PROM1 may play a role in cell differentiation, proliferation, and apoptosis. It also plays a major role as the principal regulator of disk morphogenesis and MAPK and AKT signaling pathways [69–71]. Several studies have shown that mutations in PROM1 are associated with photoreceptor degeneration in mice [69, 72, 73] and this photoreceptor degeneration is regulated through interactions between PROM1 and PCDH21 [69]. It has also been reported that PROM1 directly interacts with actin filaments (β-actin) in the protrusions of cells [69]. However, the factors associated with the expression of PROM2 remain largely unidentified. In silico studies suggest that PROM2 transcription may be regulated by several proteins, including E74A, HFH-2, Snail, and Spz1 [74, 75]. Moreover, PROM2 has been reported to be a testosterone-regulated gene in the rat ventral prostate [76], which suggests the possible hormone-mediated regulation of PROM2 expression. Another study has also reported that PROM2 is regulated by the androgen receptor (AR) [77]. Therefore, regulation of prominins by other interacting partners warrants further investigation. To identify the PPIs involving prominins, we used GeneMANIA, which compiles data on co-expression, co-localization, genetic interactions, pathways involved, physical interaction predictions, and shared protein domains. We selected the prominent functional protein partners of prominins from this analysis, as mentioned below, for further investigation. The predicted protein partners of PROM1 along with their respective genes were: prominin 2 (PROM2), gastrin-releasing peptide (GRP), zinc finger protein 157 (ZNF157), frizzled related protein (FRZB), claudin 10 (CLDN10), phosphatidylinositol-4-phosphate 5-kinase type 1 beta (PIP5K1B), cadherin-related family member 1 (CDHR1), C-X3-C motif chemokine ligand 1 (CX3CL1), and PDZ domain-containing protein (PDZD2, Fig. 5a [i]). The predicted protein partners of PROM2 along with their corresponding genes were: prominin 1 (PROM1), ETS homologous factor (EHF), family with sequence similarity 110 member C (FAM110C), sphingosine-1-phosphate phosphatase 2 (SGPP2), adhesion G protein-coupled receptor G1 (ADGRG1), PGAP2-interacting protein (CWH43), proline-rich gla protein 2 (PRRG2), tetraspanin 1 (TSPAN1), and E74-like ETS transcription factor 3 (ELF3, Fig. 5a [ii]). Thus, these predicted interacting partners of prominins may be involved in the regulation of prominin-mediated cancer progression and prognosis.

### Cross-cancer analysis of prominin mutations and copy number alterations

We analyzed genetic alterations of PROM1 in different cancers using cBioPortal and compared the results with those of other genes of interest mentioned in the preceding subsection. The database was first queried for PROM1 gene mutations in 56,250 samples from 215 studies that covered the entire set of available cancers. The gene set or pathway was altered in 310 of the queried samples, with a somatic mutation frequency of 0.6%. As shown in Fig. 5b (i), 331 mutations, including 118 duplications were detected in patients with multiple samples. The mutation sites were located between amino acids 0 and 865. Of these mutations, 243 missense mutations and 88 truncating mutations were detected. We also observed that PROM1 mutations primarily occurred in uterine cancer and spanned the prominin domain, with a hotspot in N566Ifs*29/Kfs*2. The database was also queried for PROM2 using the same settings as for PROM1. In this case, the gene set or pathway was altered in 372 of the queried samples. Therefore, the somatic mutation frequency was 0.7%, which was slightly higher than the frequency for PROM1. In total, 405 mutations, including 114 duplications, were detected, which were located between amino acids 0 and 834. Thus, the mutations in PROM2 were slightly denser than those in PROM1. We found 344 missense mutations, 55 truncations, and 6 in-frame mutations among these mutations. Unlike PROM1, PROM2 mutations primarily occurred in skin cancer. They also spanned the prominin domain, with hotspots in Q508R and R582Q/W (Fig. 5b [ii]).

Next, we analyzed mutations and CNAs in a set of genes (corresponding to functional protein partners) centered around PROM1 (Fig. 5a [i]) in 215 different cancer studies. The query was customized to select 20 different cancer studies, representing 5796 samples that contained an alteration frequency > 25%, with at least 100 samples in each dataset (Fig. 5c [i], Supplementary Table 5). Fig. 5c (i) shows that the alteration frequency ranged from 25.27 to 52.34%. The alterations occurred mostly in neuroendocrine prostate cancer (NEPC). Similarly, an analysis of mutations and CNAs in the PROM2-centered functional partner genes (Fig. 5a [ii]) showed alteration frequencies ranging from 20.2 to 44.9% (Fig. 5c [ii], Supplementary Table 6). Similar to PROM1, the PROM2-centered gene set also showed that, with the exception of deep deletions, alterations occurred mainly in NEPC (Fig. 5c, Supplementary Tables 5 and 6).

**Fig. 6:**
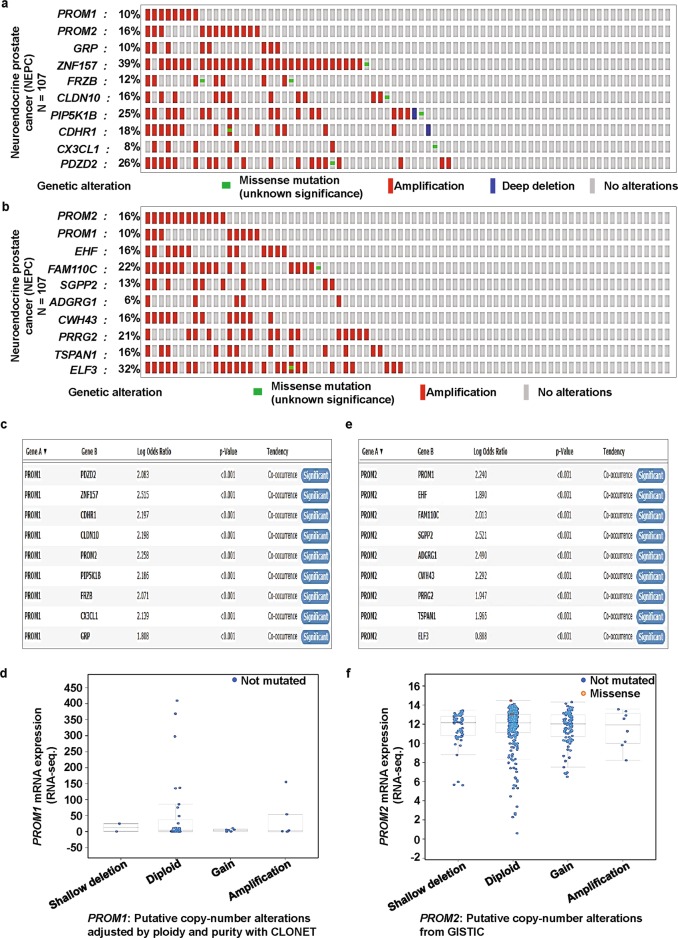
PROM1 and PROM2 gene signatures are predominantly amplified and significantly co-expressed in neuroendocrine prostate cancer (NEPC) and bladder cancer. **a** We used the Oncoprint feature of cBioPortal to determine the copy number alteration frequency of each individual gene of the PROM1 (**a**) cluster (PROM1, PROM2, GRP, ZNF157, FRZB, CLDN10, PIP5K1B, CDHR1, CX3CL1, and PDZD2) and the PROM2 (**b**) cluster (PROM2, PROM1, EHF, FAM110C, SGPP2, ADGRG1, CWH43, PRRG2, TSPAN1, and ELF3) within selected cancer subtypes. The alterations included missense mutations (green), amplifications (red), deep deletions (blue), or no alterations (grey). **c** and **d** Mutual exclusivity panel analysis revealed the co-occurrence of alterations of PROM1 gene signatures and correlations between PROM1 gene copy number and mRNA expression. The correlation between PROM1 CNAs and mRNA levels was investigated using the cBioPortal for Cancer Genomics. Data are shown for the 107 NEPC samples in which CNAs were available. The search criteria were: “neuroendocrine prostate cancer (NEPC), mutation, and putative copy-number alterations adjusted by ploidy and purity with CLONET.” The x-axis is divided according to the copy number status of the tumor and the Y-axis represents PROM1 mRNA levels. **e** and **f** Mutual exclusivity panel analysis revealed the co-occurrence of alterations of PROM2 gene signatures and correlation between PROM2 gene copy number and mRNA expression. The correlation between PROM2 CNAs and mRNA levels was investigated using cBioPortal for Cancer Genomics. Data are shown from the 402 bladder cancer (TCGA PanCan Atlas) samples in which CNAs were available. The search criteria were “Bladder cancer (TCGA PanCan Atlus), mutation, putative copy-number alterations from GISTIC, and mRNA expression *z*-Scores (U133 microarray only).” The x-axis is divided according to the copy number status of the tumor and the Y-axis represents PROM2 mRNA level

Next, we applied the OncoPrint sub-tool of cBioPortal to investigate how the genomic alterations in NEPC are distributed over various genes corresponding to the functional protein partners of prominins. For PROM1, alterations in the gene set of PROM1, PROM2, GRP, ZNF157, FRZB, CLDN10, PIP5K1B, CDHR1, CX3CL1, and PDZD2 are shown in Fig. 6a. For PROM2, alterations in the gene set of PROM2, PROM1, EHF, FAM110C, SGPP2, ADGRG1, CWH43, PRRG2, TSPAN1, and ELF3 are shown in Fig. 6b. In the PROM1 and PROM2 gene sets, the alteration percentages varied in the range of 8–39% and 6–32%, respectively, for individual genes. In both cases, alterations largely occurred due to amplifications. Notably, ZNF157 was predominantly amplified in the PROM1-centered gene set (Fig. 6a) and this gene has been shown to be epigenetically regulated in medulloblastoma [78]. In the PROM2-centered gene set, however, ELF3, a member of the E-twenty-six family of transcription factors, was predominantly amplified (Fig. 6b). It plays a key role in β-catenin signaling in colorectal cancer and thus, has potential prognostic and therapeutic significance [79]. The genomic alterations in other cancer types were distributed over various genes corresponding to functional protein partners of prominins. These are summarized in Supplementary Tables 7 and 8.

To determine whether each member in the set of genes corresponding to the functional protein partners of PROM1 were significantly correlated, we used the co-occurrence analysis sub-tool of cBioPortal, which is based on Fisher’s exact test. This analysis confirmed the statistical significance of the co-occurrence of alterations in PROM1 and each gene in the associated set (Fig. 6c). A similar analysis was performed for PROM2, which also shows the statistically significant co-occurrence of alterations of each pair of PROM2 and its partner genes (Fig. 6e). An analysis of the corresponding levels of expression associated with the mutation status of prominins showed that deletions of both PROM1 and PROM2 correlated with increased mRNA expression (Figs. 6d and f).

**Fig. 7:**
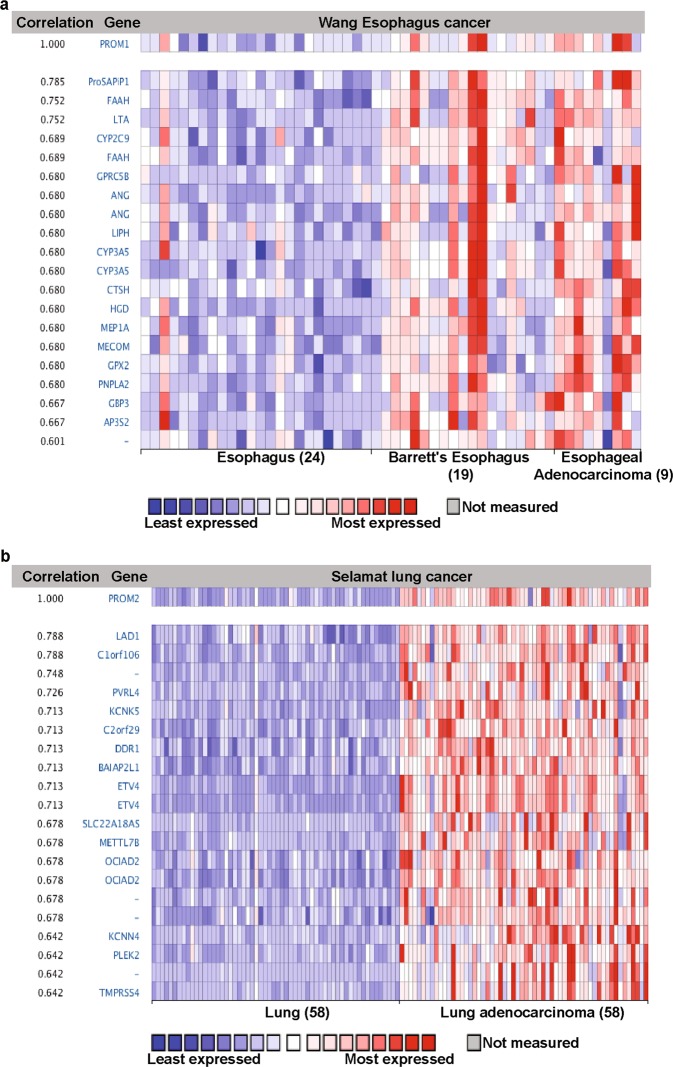
Co-expression profile of PROM1 and PROM2 in esophageal and lung adenocarcinoma. **a** PROM1 is co-expressed with the indicated genes across a panel of nine esophageal adenocarcinomas, 19 Barrett’s esophagus samples, and 24 normal samples. **b** PROM2 is co-expressed with the indicated genes across a panel of 58 lung adenocarcinomas and 58 normal samples. Bar length represents the significance and negative logarithm of the enrichment *p*-value

The Oncomine database provides an important list of co-expressed genes, which may help to identify the pathways involved. Co-expression analysis showed that PROM1 was significantly co-expressed with ProSAPiP1, FAAH, and LTA in esophageal cancer (Fig. 7a). In liver cancer PROM1 was more strongly co-expressed with a larger number of genes, including CFTR, KRT7, ANXA3, TACSTD2, and FZD1 (Supplementary Fig. S8). ANXA3 encodes the annexin 3 protein, which can interact with acidic phospholipids in a calcium-dependent manner. Its dysregulation has a potential role in tumorigenesis [80]. Another study reported that the upregulation of TACSTD2 regulates breast cancer invasiveness and subsequently, correlates with poor prognosis [81]. In contrast, PROM2 was significantly co-expressed with LAD1, C1ORF106, PVRL4, and KCNK5 in lung cancer (Fig. 7b). Previous studies have reported that LAD1, C1ORF106, PVRL4, and KCNK5 are upregulated in cancer cells [82–85]. Furthermore, LAD1 has been reported as a filament-binding regulator and also regulates EGF signaling-mediated breast cancer tumorigenesis [82]. In ovarian cancer, PROM2 is slightly co-expressed with CARD14 (Supplementary Fig. S9). Thus, the findings presented here, in combination with those of previous studies, provide ample evidence that prominin expression may be involved in cancer progression and prognosis by associating with co-expressed genes.

### Genes correlated with prominin genes and their functional GO and pathways

**Fig. 8:**
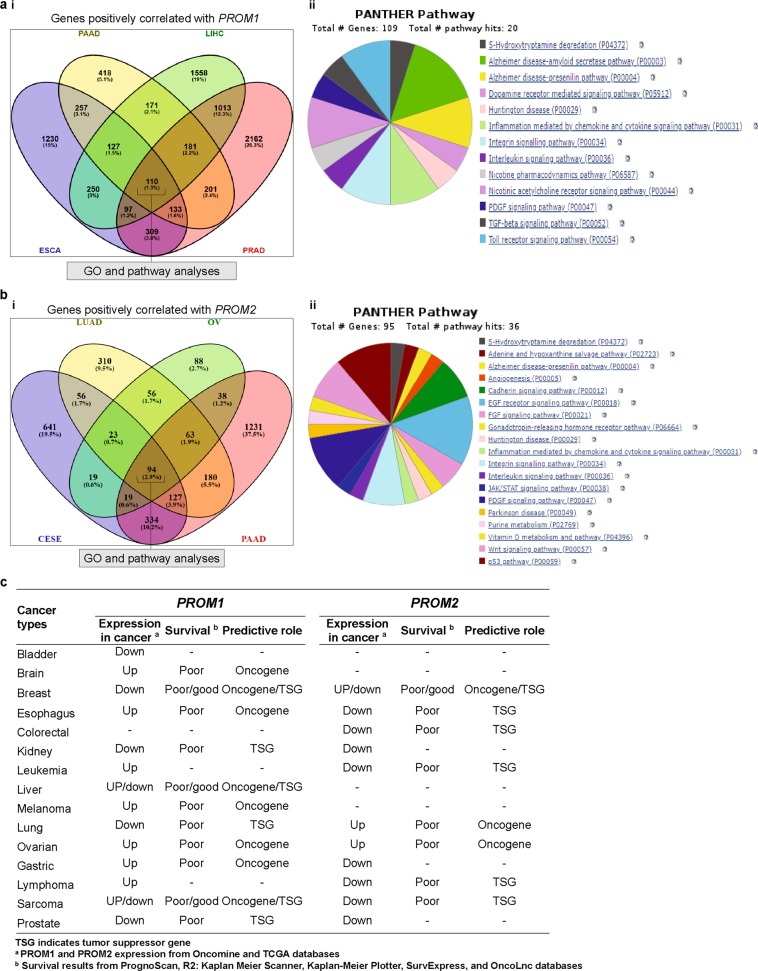
Analysis of genes that positively correlated with PROM1 and PROM2 and their predicted pathway analysis using PANTHER. **a** Venn diagram of genes that positively correlated with PROM1, showing coincident genes in ESCA, PAAD, LIHC, and PRAD (**i**). Pathway analysis using PANTHER and subsequent classification based on their pathways (**ii**). **b** Venn diagram of genes that positively correlated with PROM2, showing coincident genes in CESE, LUAD, OV, and PAAD (**i**). Pathway analysis using PANTHER and subsequent classification based on their pathways (**ii**). **c** Summary of predictive value of PROM1 and PROM2 in different cancers, based on systematic genetic analyses

To identify genes that correlate with PROM1 and PROM2 expression in selected cancers, we performed a systematic analysis using the R2 platform, as outlined below. Two different sets were selected, each containing four tumor types for both PROM1 and PROM2, based on their high levels of expression in those cancers. From the advanced dataset selection panel of R2, we first selected the TCGARS platform as the expression data for a particular cancer. Based on the degree of expression, we individually considered the following four tumor types for PROM1: esophageal, liver, pancreatic, and prostate tumors. Next, we made a query to identify the list of genes that correlated with PROM1 in these tumor types, individually using Bonferroni correction and a *p*-value < 0.01 for each case. We observed that 2513, 1598, 3507, and 4206 genes positively correlated with PROM1 expression in esophageal (ESCA), pancreatic (PAAD), liver (LIHC), and prostate (PRAD) cancers, respectively. One hundred and ten genes (hereafter referred to as “PROM1-correlated gene cluster”) that positively correlated with PROM1 were common among all four cancer types considered in this analysis (Fig. 8a [i]). There were no common genes detected that negatively correlated with PROM1 in these four tumors (Supplementary Fig. S10). We performed a similar analysis for PROM2, assessing cervical (CESE), lung (LUAD), ovarian (OV), and pancreatic (PAAD) tumors. As discussed below, we analyzed a different set of cancer types for PROM2 because high expression levels of this gene were detected in these cancers. Compared to PROM1, fewer genes correlated positively with PROM2 in each cancer type and 94 genes (hereafter referred to as “PROM2-correlated gene cluster”) were common in all four cancers analyzed (Fig. 8b [i]). In addition, a short list of three genes was identified that negatively correlated with PROM2 in all cancers analyzed (Supplementary Fig. S10). Finally, as we compared the genes that correlated with PROM1 or PROM2 in each individual tumor type, we observed a substantial number of commonly correlated genes with the same correlation direction and we observed no commonly correlated genes with the reverse correlation direction. This suggested that PROM1 and PROM2 participate in many common processes, depending on the tissue.

Next, we performed GO and pathway analyses for the PROM1/PROM2-correlated gene clusters using the web-based classification system, PANTHER. We made a separate query for the functional classification of each of the correlated genes and presented the four important ontologies associated with them. Each of the ontologies consisted of multiple sub-ontologies. We showed that binding, catalytic, and transporter activities were the main molecular functions associated with the PROM1-correlated gene cluster (Supplementary Fig. S11a [i]), whereas binding and catalytic activity were the major molecular functions associated with the PROM2-correlated gene cluster (Supplementary Fig. S11b [i]). In addition, the major biological process associated with both PROM1 and PROM2-correlated gene clusters was “Cellular process” (Supplementary Figs S11a [ii], S9b [ii]). “Cell part” and “Membrane” were the two key cellular components associated with both PROM1- and PROM2-correlated gene clusters (Supplementary Figs S11a [iii], S11b [iii]). The PROM1-correlated gene cluster affected 20 pathways, whereas the PROM2-correlated gene cluster affected 36 pathways and was involved in more diverse roles (see Figs. 8a [ii], 8b [ii]). The Alzheimer disease-amyloid secretase pathway was the most significant pathway associated with the PROM1-correlated gene, whereas the EGF receptor signaling pathway was the key player in the PROM2-correlated gene cluster. However, seven common pathway classes occurred in both clusters, including those involved in 5-hydroxytryptamine degradation, Alzheimer disease-presenilin, chemokine and cytokine signaling, integrin signaling, interleukin signaling, and PDGF signaling. In summary, our results suggested that, although PROM1 and PROM2 participate in the regulation of different pathways, they demonstrate similar correlations in certain signaling pathways, depending on the tissue.

## Discussion

Prominins have been shown to contribute to the generation and development of various cancers [4, 6, 86]. *PROM1* is reported to be a regulator of stem cell activation by modifying microvillar architecture and dynamics and ciliary dynamics [12, 18]. *PROM2* can cause cellular protrusions and enhance phosphorylation of caveolin-1 to promote endocytosis [23]. In addition, several studies have shown that *PROM1* and *PROM2*-targeted therapies are promising for the prevention of tumor development [14, 87]. However, the role of these prominin family members in the development of human cancers is still not understood. To determine the utility of *PROM1* and *PROM2* as markers of cancer prognosis, we performed a systematic data analysis of numerous gene expression datasets with clearly defined distinguishing parameters between cancer and normal tissues. *PROM1* and *PROM2* were found to be differentially expressed in cancer and normal tissues. The extent of their expression also differed depending on the tissue, according to Oncomine and GEPIA-based transcription analysis. Analysis of Oncomine data showed that *PROM1* was upregulated in leukemia and esophageal, liver, and ovarian cancers, but downregulated in other cancer types, including kidney cancer. In contrast, *PROM2* was upregulated in myeloma and breast, lung, and ovarian cancers and downregulated in colon, esophageal, gastric, kidney, and prostate cancers.

To improve our understanding of the value of prominins as prognostic markers, we next investigated the association between their expression levels and OS in various cancers. The prognostic value of *PROM1* and *PROM2* mRNA expression was assessed using PrognoScan, R2, Kaplan-Meier plotter, SurvExpress, and OncoLnc. In general, high *PROM1* expression was associated with poor survival in mixed Ewing sarcoma and brain, esophageal, gastric, liver, and ovarian cancers. However, owing to contradictory results from different analyses, its role in liver cancer was not clear. Our observations are in agreement with the results of several previous studies. For example, *PROM1* upregulation has been reported in ovarian cancer [88, 89] and targeting this gene retards ovarian cancer development in an *in vivo* model [90]. Furthermore, *PROM1* has been identified as a stem cell marker in esophageal and breast cancers [5, 56]. Next, we observed that *PROM2* upregulation was associated with poor OS in lung cancer; however, owing to contradictory results, its role in breast cancer was not clear and the receptor status of breast cancer cells also had some effect on patient survival. Any significant deviation (either high or low) in *PROM2* expression can be considered as a valid prognostic marker of low survival rates in patients with ovarian cancer. Thus, based on mRNA expression and clinical data from Oncomine and TCGA, we conclude that *PROM1* has as an oncogenic role in brain, esophageal, ovarian, and gastric cancers and melanoma and *PROM2* shows oncogenic behavior in lung and ovarian cancers (Fig. 8c). To assess the impact of *PROM1*/*PROM2* co-expression on various cancers, we performed several multivariate survival analyses on OncoLnc, using TCGA data. The co-expression (high/high), partial co-expression (low/high), and lack of co-expression (low/low) of *PROM1*/*PROM2* were associated with skin, KIRC, and KIRP cancers, respectively. The results suggested that the co-expression of *PROM1* and *PROM2* may impact clinical outcomes in patients with certain types of cancers.

Tumorigenesis is a multi-step process that leads to the development of a tumor. It is primarily influenced by four major factors - somatically acquired genetic, epigenetic, transcriptomic, and proteomic alterations [91, 92]. Somatic loss-of-function or gain-of-function alterations occur in particular genomic regions involved in potential inhibitory or carcinogenic effects, respectively [93, 94]. Therefore, we used cBioPortal to determine human cancers with CNAs and mutations in prominin genes. Missense and truncating mutations predominantly occurred within protein-coding sequences. Mutations in *PROM1* were mainly missense and truncating mutations, including p.R373C, p.Y452fsX12, p.G614fsX626, and p.Q576X in the prominin domain, which are associated with human inherited diseases [95–98]. A recent study reported the identification of a point mutation at p.S281R (serine [S] changed to arginine [R] due the mutation of thymine [T] to guanine [G]) in the prominin domain of the *PROM1* protein in 8/555 lung cancer patients [99]. However, mutations in the *PROM2* protein have not been well studied. In our systematic analysis, we also found several missense and truncating mutations within *PROM1* and *PROM2* protein-coding sequences, especially in the prominin domain, in various cancer types. These results are yet to be experimentally validated. Specifically, *PROM1* mutations mainly occurred in uterine pan-cancer and consisted of several frame-shift insertion or deletion mutations in a hotspot at position p.N566Ifs*29/Kfs*2 in the prominin domain. *PROM2* mutations were mainly found in skin cancers and predominantly consisted of point mutations of a hotspot at position p.Q508R and p.R582Q/W in the prominin domain. These mutations have a potential role in the regulation of cancer progression and prognosis, but this is yet to be confirmed. PPIs trigger the majority of biological processes, including signaling and disease development [100]. Thus, understanding the factors that modulate PPIs is important. Therefore, we first identified the top ten significantly correlated functional protein partners of *PROM1* and *PROM2* and constructed their interaction network using GeneMANIA. With a cross-cancer viewpoint for both, as Subsequent analysis using cBioPortal showed that genetic alterations of the ten genes in the *PROM1* and *PROM2* signatures, mainly occurred in NEPC, with alteration frequencies of 25.27–52.34% and 20.2–44.9%, respectively. Genomic alterations in NEPC were differentially distributed over the set of genes, with individual gene alterations occurring at frequencies of 8 to 39% for *PROM1*-related genes and 6–32% for *PROM2*-related genes.

A high degree of correlation between *PROM1* and *PROM2* gene expression indicates that they have either have comparable roles or are involved in the same biochemical processes and may be co-regulated [101]. Therefore, the R2 genomics platform was used to identify genes correlated with *PROM1* and *PROM2* in certain cancers in which these prominins are highly expressed. For *PROM1*, a large number of positively correlated genes were detected in esophageal, pancreatic, liver, and prostate cancers. Of those genes, 110 were common in all cancers. For *PROM2*, a relatively fewer number of correlated genes were detected in cervical, lung, ovarian, and pancreatic tumors, among which 94 genes were common in all cancers. Next, to determine the shared role of these correlated genes in the above cancers, we used the online tool, PANTHER, to perform GO and pathway analyses. Despite the existence of seven common pathways, *PROM2*-correlated genes demonstrated more varied characteristics than *PROM1*-correlated genes (20 vs. 36 pathways). This correlation analysis suggested that *PROM1* and *PROM2* perform different functions with respect to pathway regulation; however, they may have some similar functions in certain signaling pathways, in certain cancer types.

In summary, we investigated the expression, mutation, and copy number alteration patterns of *PROM1* and *PROM2* genes and assessed their prognostic significance through a systematic data analysis, using publicly available expression and clinical data. This analysis was able to predict the expression status of prominins for various cancer types. These data suggest that prominin expression may be translated into clinical practice and that their co-expression may impact clinical outcomes in patients with certain types of cancers. In addition, this study found that some functional protein partners of prominins show high frequencies of genomic alterations in certain cancer types. For example, amplifications were predominantly found in *ZNF157* and *ELF3* genes among the *PROM1*- and *PROM2*-centered gene sets, respectively, in NEPC. Therefore, investigation of the combined roles of *PROM1* and ZNF157 and *PROM2* and ELF3 are important topics for future research. Moreover, we propose that effects of *PROM1* and *PROM2* on cancer progression and prognosis, may be more prominent when associated with their respective co-expressed genes, such as ANXA3 and TACSTD2 for *PROM1* and LAD1 and C1ORF106 for *PROM2*. Furthermore, in this study, we demonstrated that *PROM1* and *PROM2* perform different functions with respect to pathway regulation.

### Concluding remarks

In this study, we focused on data mining approaches to investigate the expression, mutation, and CNA of prominins with respect to clinical outcomes in various cancers. Our results demonstrated that *PROM1* and *PROM2* expression differentially modulate the clinical outcomes of cancer patients. While the former can act as a therapeutic target for certain cancers, including esophageal, liver, and ovarian cancer, the latter can be considered as an oncogenic marker for ovarian and lung cancers. Meanwhile, both *PROM1* and *PROM2* are potential targets for skin and kidney cancers. PPI and co-expression analyses of prominins may also predict the probable underlying signaling mechanisms associated with prominins in certain cancers. In summary, these multiomics findings are expected to improve our understanding of the relationship between prominin expression and clinical prognosis. They also provide new insights into the molecular mechanisms involved in cancer and thus, will assist in transforming genomic knowledge into clinical practice. Hence, the specific roles, detailed molecular mechanisms, and clinical significance of prominins in cancer progression and prognosis deserve further investigation.

## Supplementary information

Supplementary Material
