# Supplementary material for: PROM1 and PROM2 expression differentially modulates clinical prognosis of cancer: a multiomics analysis
Source: Cancer Gene Ther. 2019 Jun 5;27(3):147–67. doi: 10.1038/s41417-019-0109-7 (PMC7170805; doi:10.1038/s41417-019-0109-7)
Supplement: Supplementary file 1 — Supplementary Material [file 41417_2019_109_MOESM1_ESM.docx]

**Supplementary Materials**

*PROM1* and *PROM2* Expression Differentially Modulates Clinical Prognosis of Cancer: A Multiomics Analysis

Subbroto Kumar Saha^1, †,^*, S.M. Riazul Islam^2,†^, Kyung-Sup Kwak^3^, Md Shahedur Rahman^4^, and Ssang-Goo Cho^1,^ *

^1^Department of Stem Cell and Regenerative Biotechnology, Konkuk University, 120 Neungdong-ro, Gwangjin-gu, Seoul 05029, Republic of Korea; ^2^Department of Computer Science and Engineering, Sejong University, 209, Neungdong-ro, Gwangjin-gu, Seoul 05006, Republic of Korea; ^3^School of Information and Communication Engineering, Inha University, 100, Inha-ro, Nam-gu, Incheon 22212, Republic of Korea; ^4^Department of Genetic Engineering and Biotechnology, Jashore University of Science and Technology, Jashore 7408, Bangladesh;

† These authors contributed equally to this work: S.K. Saha and S.M.R. Islam (Co-first authors).

* Correspondence: Subbroto Kumar Saha (subbroto@konkuk.ac.kr; Tel.: +82-2-450-0574) and Ssang-Goo Cho (ssangoo@konkuk.ac.kr; Tel.: +82-2-450-4207; Fax: +82-2-444-4207 (S.-G.C. & S.K.S.).

Department of Stem Cell and Regenerative Biotechnology, Konkuk University, 120 Neungdong-Ro, Seoul 05029, Republic of Korea

**Running title: Multiomics Analysis of *PROM1* and *PROM2* Expression**

**Supplementary Tables**

**Supplementary Table S1.** *PROM1* expression in various cancers from the Oncomine database.

| **Cancer** | **Cancer subtype** | **p-value** | **Fold change** | **Rank (%)** | **Sample** | **Reference** |
| --- | --- | --- | --- | --- | --- | --- |
| Bladder | Superficial Bladder Cancer | 6.83E-12 | -2.598 | 3 | 256 | [[1](#_ENREF_1)] |
| Brain | Glioblastoma | 3.69E-06 | 2.667 | 6 | 84 | [[2](#_ENREF_2)] |
| Breast | Mucinous breast Carcinoma | 1.81E-16 | -5.213 | 6 | 2136 | [[3](#_ENREF_3)] |
| Oesophagus | Barrett's oesophagus | 3.28E-23 | 44.008 | 1 | 118 | [[4](#_ENREF_4)] |
|  | Oesophageal Adenocarcinoma | 4.82E-18 | 12.106 | 1 | 118 | [[4](#_ENREF_4)] |
|  | Barrett's oesophagus | 3.12E-08 | 5.183 | 1 | 48 | [[5](#_ENREF_5)] |
| Kidney | Clear cell sarcoma of the kidney | 9.31E-06 | -5.859 | 2 | 35 | [[6](#_ENREF_6)] |
|  | Non-hereditary clear cell renal cell carcinoma | 8.27E-06 | -6.671 | 7 | 70 | [[7](#_ENREF_7)] |
|  | Chromophobe renal cell carcinoma | 6.41E-05 | -3.584 | 9 | 92 | [[8](#_ENREF_8)] |
| Leukaemia | Pro-B acute lymphoblastic leukaemia | 1.71E-14 | 13.222 | 3 | 1152 | [[9](#_ENREF_9)] |
|  | Acute myeloid leukaemia | 1.54E-31 | 2.132 | 5 | 1152 | [[9](#_ENREF_9)] |
|  | Acute myeloid leukaemia | 1.01E-30 | 2.051 | 2 | 2096 | [[9](#_ENREF_9)] |
|  | Pro-B acute lymphoblastic leukaemia | 6.93E-25 | 9.294 | 3 | 2096 | [[9](#_ENREF_9)] |
| Liver | Cirrhosis | 1.54E-07 | 6.499 | 1 | 75 | [[10](#_ENREF_10)] |
|  | Cirrhosis | 9.64E-23 | 3.696 | 1 | 115 | [[11](#_ENREF_11)] |
|  | Hepatocellular carcinoma | 1.99E-12 | -2.279 | 5 | 197 | [[12](#_ENREF_12)] |
| Melanoma | Benign melanocytic skin nevus | 7.42E-05 | -3.566 | 4 | 70 | [[13](#_ENREF_13)] |
| Other | Testicular embryonal carcinoma | 2.01E-06 | 4.384 | 1 | 30 | [[14](#_ENREF_14)] |
|  | Seminoma, NOS | 1.27E-07 | 4.658 | 5 | 107 | [[15](#_ENREF_15)] |
| Ovarian | Ovarian mucinous adenocarcinoma | 9.52E-08 | 2.285 | 2 | 103 | [[16](#_ENREF_16)] |
|  | Ovarian clear cell adenocarcinoma | 4.67E-05 | 2.26 | 5 | 103 | [[16](#_ENREF_16)] |
| Gastric | Gastrointestinal stromal tumour | 1.14E-06 | 6.013 | 2 | 90 | [[17](#_ENREF_17)] |

**Supplementary Table S2.** *PROM2* expression in various cancers from the Oncomine database.

| **Cancer** | **Cancer subtype** | **p-value** | **Fold change** | **Rank (%)** | **Sample** | **Reference** |
| --- | --- | --- | --- | --- | --- | --- |
| Breast | Mucinous breast carcinoma | 2.33E-05 | 3.288 | 2 | 593 | TCGA |
|  | Invasive ductal and lobular carcinoma | 3.82E-07 | 2.256 | 2 | 593 | TCGA |
|  | Male breast carcinoma | 1.28E-05 | 2.302 | 3 | 593 | TCGA |
|  | Invasive ductal breast Carcinoma | 7.27E-05 | -38.28 | 2 | 22 | [[18](#_ENREF_18)] |
| Colorectal | Colon carcinoma | 4.79E-14 | -22.809 | 1 | 40 | [[19](#_ENREF_19)] |
|  | Colon carcinoma | 9.28E-06 | -17.014 | 8 | 40 | [[19](#_ENREF_19)] |
|  | Colon adenocarcinoma | 5.00E-19 | -4.086 | 1 | 105 | [[20](#_ENREF_20)] |
|  | Recto sigmoid adenocarcinoma | 2.49E-06 | -4.79 | 2 | 105 | [[20](#_ENREF_20)] |
|  | Cecum adenocarcinoma | 7.62E-07 | -3.195 | 2 | 105 | [[20](#_ENREF_20)] |
|  | Rectal adenocarcinoma | 3.28E-05 | -4.481 | 3 | 105 | [[20](#_ENREF_20)] |
|  | Colon mucinous adenocarcinoma | 2.57E-05 | -4.372 | 6 | 105 | [[20](#_ENREF_20)] |
|  | Colorectal carcinoma | 6.09E-21 | -5.582 | 1 | 82 | [[21](#_ENREF_21)] |
|  | Colorectal carcinoma | 4.13E-14 | -4.107 | 1 | 105 | [[19](#_ENREF_19)] |
|  | Colon adenocarcinoma | 4.16E-17 | -3.905 | 5 | 237 | TCGA |
|  | Rectal adenocarcinoma | 2.29E-15 | -4.291 | 7 | 237 | TCGA |
|  | Rectal adenocarcinoma | 1.59E-19 | -3.376 | 7 | 130 | [[22](#_ENREF_22)] |
| Oesophagus | Oesophageal adenocarcinoma | 4.77E-12 | -3.499 | 8 | 118 | [[4](#_ENREF_4)] |
| Gastric | Diffuse gastric adenocarcinoma | 6.32E-06 | -4.169 | 3 | 90 | [[17](#_ENREF_17)] |
|  | Gastrointestinal stromal tumour | 2.91E-11 | -17.055 | 1 | 90 | [[17](#_ENREF_17)] |
| Kidney | Renal Wilms tumour | 5.26E-05 | -20.805 | 1 | 67 | [[23](#_ENREF_23)] |
|  | Renal oncocytoma | 2.90E-05 | -27.356 |  | 67 | [[23](#_ENREF_23)] |
|  | Clear cell renal cell carcinoma | 2.44E-07 | -14.427 | 1 | 67 | [[23](#_ENREF_23)] |
|  | Papillary renal cell carcinoma | 4.97E-07 | -33.444 | 2 | 67 | [[23](#_ENREF_23)] |
| Lung | Lung adenocarcinoma | 1.30E-25 | 5.069 | 1 | 116 | [[24](#_ENREF_24)] |
|  | Lung adenocarcinoma | 3.67E-18 | 5.675 | 1 | 246 | [[25](#_ENREF_25)] |
|  | Squamous cell lung carcinoma | 6.68E-05 | 4.698 | 2 | 73 | [[26](#_ENREF_26)] |
|  | Squamous cell lung carcinoma | 3.10E-08 | 3.701 | 8 | 156 | [[27](#_ENREF_27)] |
| Lymphoma | Anaplastic large cell lymphoma | 3.20E-06 | -2.154 | 6 | 60 | [[28](#_ENREF_28)] |
| Myeloma | Monoclonal gammopathy of undetermined significance | 1.86E-06 | 2.939 | 5 | 78 | [[29](#_ENREF_29)] |
| Melanoma | Skin basal cell carcinoma | 2.53E-06 | -2.052 | 1 | 87 | [[30](#_ENREF_30)] |
| Ovarian | Ovarian serous adenocarcinoma | 6.76E-07 | 2.935 | 1 | 50 | [[31](#_ENREF_31)] |
|  | Ovarian serous adenocarcinoma | 6.55E-05 | 12.355 | 8 | 53 | [[32](#_ENREF_32)] |
| Prostate | Prostate carcinoma | 5.99E-06 | -2.026 | 7 | 122 | [[33](#_ENREF_33)] |

**Supplementary Table S3.** Association of *PROM1* expression and survival of cancer patients (PrognoScan database).

| **Cancer type** | **Endpoint** | **N** | **Dataset** | **Probe ID** | **Cox P-value** | **HR [95% CI^low^- CI^upp^]** |
| --- | --- | --- | --- | --- | --- | --- |
| Soft tissue cancer | Distant recurrence Free survival | 140 | GSE30929 | 204304_s_at | 0.0001 | 1.54 [1.19 - 1.99] |
| Brain cancer | Overall survival | 70 | GSE7696 | 204304_s_at | 0.0132 | 1.28 [1.05 - 1.55] |
| Prostate cancer | Overall survival | 281 | GSE16560 | DAP3_4133 | 0.0186 | 0.80 [0.66 - 0.96] |
| Brain cancer | Overall survival | 50 | MGH-glioma | 41470_at | 0.0189 | 1.44 [1.06 - 1.96] |
| Brain cancer | Overall survival | 74 | GSE4412-GPL96 | 204304_s_at | 0.022 | 1.60 [1.07 - 2.40] |
| Lung cancer | Overall survival | 104 | jacob-00182-MSK | 204304_s_at | 0.0229 | 0.69 [0.51 - 0.95] |
| Breast cancer | Relapse-free survival | 204 | GSE12276 | 204304_s_at | 0.0269 | 1.09 [1.01 - 1.17] |
| Brain cancer | Overall survival | 77 | GSE4271-GPL96 | 204304_s_at | 0.0342 | 1.36 [1.02 - 1.80] |
| Skin cancer | Overall survival | 38 | GSE19234 | 204304_s_at | 0.0387 | 1.52 [1.02 - 2.26] |
| Breast cancer | Distant metastasis- free survival | 200 | GSE11121 | 204304_s_at | 0.0454 | 1.27 [1.00 - 1.60] |
| Breast cancer | Distant metastasis- free survival | 136 | GSE12093 | 204304_s_at | 0.048 | 0.71 [0.50 - 1.00] |

**Supplementary Table S4.** Association of *PROM2* expression and survival of cancer patients (PrognoScan database).

| **Cancer type** | **Endpoint** | **N** | **Dataset** | **Probe ID** | **Cox P-value** | **HR [95% CI^low^- CI^upp^]** |
| --- | --- | --- | --- | --- | --- | --- |
| Breast cancer | Relapse-free survival | 60 | GSE1379 | 12323 | 0.004 | 12.52 [2.24 - 70.04] |
| Breast cancer | Relapse-free survival | 204 | GSE12276 | 1562378_s_at | 0.0122 | 0.80 [0.67 - 0.95] |
| Breast cancer | Relapse-free survival | 60 | GSE1378 | 1259 | 0.0189 | 2.41 [1.16 - 5.03] |
| Breast cancer | Relapse-free survival | 60 | GSE1379 | 1259 | 0.0195 | 1.82 [1.10 - 3.01] |
| Lung cancer | Relapse-free survival | 138 | GSE8894 | 239528_at | 0.034 | 2.00 [1.05 - 3.78] |
| Eye cancer | Distant metastasis-free survival | 63 | GSE22138 | 1562378_s_at | 0.0469 | 0.00 [0.00 - 0.79] |
| Blood cancer | Disease-specific survival | 559 | GSE2658 | 239528_at | 0.0498 | 0.70 [0.49 - 1.00] |

**Supplementary Table S5.** Alteration frequency of a ten-gene signature (*PROM1, PROM2, GRP, ZNF157, FRZB, CLDN10, PIP5K1B, CDHR1, CX3CL1,* and *PDZD2*) in various cancers (cBioPortal web).

| **Cancer** | **Data source** | **N** | **Frequency  (%)** | **Mutation % (N)** | **Amplification % (N)** | **Deletion % (N)** | **Multiple alterations % (N)** | **Fusion % (N)** |
| --- | --- | --- | --- | --- | --- | --- | --- | --- |
| NEPC | Trento/Cornell/Broad 2016 | 107 | 52.34 | 4.67 (5) | 39.25 (42) | 4.67 (5) | 3.74 (4) | - |
| Melanoma | TCGA PanCan Atlas | 365 | 42.42 | 36.09 (131) | 4.41 (16) | 0.28 (1) | 1.65 (6) | - |
| Lung Squ. | TCGA | 178 | 37.08 | 11.8 (21) | 12.36 (22) | 6.18 (11) | 6.74 (12) | - |
| Prostate | FHCRC, 2016 | 136 | 36.76 | 7.35 (10) | 22.06 (30) | 5.88 (8) | 1.47 (2) | - |
| Stomach | TCGA pub | 287 | 36.59 | 18.47 (53) | 7.67 (22) | 8.36 (24) | 2.09 (6) | - |
| Melanoma | TCGA | 287 | 35.89 | 26.83 (77) | 5.92 (17) | 1.39 (4) | 1.74 (5) | - |
| Pancreas | UTSW | 109 | 35.78 | - | 11.93 (13) | 22.02 (24) | 1.83 (2) | - |
| Oesophagus | TCGA | 184 | 33.7 | 11.41 (21) | 10.87 (20) | 8.15 (15) | 3.26 (6) | - |
| Lung Squ. | TCGA PanCan Atlas | 469 | 33.48 | 15.14 (71) | 10.23 (48) | 4.69 (22) | 3.41 (16) | - |
| Ovarian | TCGA | 311 | 31.51 | 2.89 (9) | 22.19 (69) | 4.82 (15) | 1.61 (5) | - |
| CCLE | Novartis/Broad 2012 | 881 | 30.99 | 2.16 (13) | 15.44 (136) | 12.71 (112) | 0.68 (6) | - |
| Stomach | TCGA | 393 | 30.79 | 14.25 (56) | 8.4 (33) | 6.36 (25) | 1.78 (7) | - |
| Bladder | TCGA, 2014 | 127 | 30.71 | 11.02 (14) | 11.02 (14) | 3.15 (4) | 5.51 (7) | - |
| Stomach/Oesophageal | TCGA | 265 | 30.57 | 5.28 (14) | 12.83 (34) | 9.06 (24) | 3.4 (9) | - |
| Bladder | TCGA | 127 | 29.92 | 9.45 (12) | 11.02 (14) | 3.15 (4) | 6.3 (8) | - |
| Uterine | TCGA PanCan | 509 | 29.86 | 20.83 (106) | 6.08 (34) | 1.57 (8) | 0.79 (4) | - |
| NSCLC | TCGA, 2016 | 1144 | 29.55 | 13.02 (149) | 9.44 (108) | 3.5 (40) | 3.58 (41) | - |
| Sarcoma | TCGA | 243 | 29.22 | 5.35 (13) | 18.93 (46) | 4.12 (10) | 0.82 (2) | - |
| Lung Squ. | TCGA pub | 178 | 28.65 | 15.73 (28) | 5.62 (10) | 3.93 (7) | 3.37 (6) | - |
| Oesophagus | TCGA PanCan | 182 | 28.57 | 7.69 (14) | 8.79 (16) | 7.69 (14) | 3.85 (7) | 0.55 (1) |
| Stomach | TCGA PanCan | 434 | 28.11 | 14.06 (61) | 6.45 (28) | 5.76 (25) | 1.61 (7) | 0.23 (1) |
| BLCA | TCGA, 2017 | 408 | 27.45 | 8.82 (36) | 9.8 (40) | 3.43 (14) | 5.39 (22) | - |
| Lung adeno. | TCGA | 230 | 27.39 | 8.26 (19) | 13.04 (30) | 3.91 (9) | 2.17 (5) | - |
| Ovarian | TCGA PanCan | 398 | 26.88 | 3.77 (15) | 16.83 (67) | 4.27 (17) | 2.01 (8) | - |
| Lung adeno. | TCGA PanCan | 507 | 26.82 | 11.83 (60) | 8.28 (42) | 3.16 (16) | 3.35 (17) | 0.2 (1) |
| The MBC project | WAGLE, 2017 | 103 | 26.21 | 7.77 (8) | 10.68 (11) | 7.77 (8) | - | - |
| Lung adeno. | TCGA pub | 230 | 26.09 | 8.26 (19) | 11.74 (27) | 3.84 (8) | 2.61 (6) | - |
| Melanoma | Broad | 121 | 25.62 | 25.62 (31) | - | - | - | - |
| Bladder | TCGA PanCan | 4.6 | 25.62 | 10.1 (41) | 8.62 (35) | 3.2 (13) | 3.69 (15) | - |
| Lung adeno. | Broad | 182 | 25.27 | 12.64 (23) | 7.14 (13) | 3.3 (6) | 2.2 (4) | - |

**Supplementary Table S6.** Alteration frequency of a ten-gene signature (*PROM2, PROM1, EHF, FAM110C, SGPP2, ADGRG1, CWH43, PRRG2, TSPAN1,* and *ELF3*) in various cancers (cBioPortal web).

| **Cancer** | **Data source** | **N** | **Frequency  (%)** | **Mutation % (N)** | **Amplification % (N)** | **Deletion % (N)** | **Multiple alterations % (N)** | **Fusion % (N)** |
| --- | --- | --- | --- | --- | --- | --- | --- | --- |
| NEPC | Trento/Cornell/Broad 2016 | 107 | 44.86 | 0.93 (1) | 42.99 (46) | - | 0.93 (1) | - |
| The MBC project | WAGLE, 2017 | 103 | 42.72 | 0.97 (1) | 35.92 (37) | 5.86 (6) | - | - |
| BLCA | TCGA, 2017 | 408 | 33.09 | 14.95 (61) | 10.29 (42) | 3.92 (16) | 3.92 (16) | - |
| Bladder | TCGA PanCan Atlas | 406 | 32.51 | 17 (69) | 8.62 (35) | 3.69 (15) | 2.96 (12) | 0.25 (1) |
| Melanoma | TCGA PanCan Atlas | 363 | 32.23 | 24.79 (90) | 4.96 (18) | - | 2.2 (8) | 0.28 (1) |
| Pancreas | UTSW | 109 | 32.11 | 0.92 (1) | 26.69 (28) | 3.67 (4) | 1.83 (2) | - |
| Bladder | TCGA | 127 | 31.5 | 12.6 (16) | 11.81 (15) | 3.94 (5) | 3.15 (4) | - |
| Breast | METABRIC | 2051 | 31.01 |  | 30.28 (621) | 0.73 (15) |  | - |
| Bladder | TCGA, 2014 | 127 | 30.71 | 12.6 (16) | 10.24 (13) | 4.72 (6) | 3.15 (4) | - |
| Melanoma | TCGA | 287 | 28.92 | 17.77 (51) | 7.32 (21) | 0.7 (2) | 3.14 (9) | - |
| Ovarian | TCGA | 311 | 27.97 | 1.93 (6) | 23.15 (72) | 2.25 (7) | 0.64 (2) | - |
| Oesophagus | TCGA | 184 | 26.63 | 10.87 (20) | 13.04 (24) | 1.63 (3) | 1.09 (2) | - |
| Uterine | TCGA PanCan | 509 | 26.13 | 15.72 (80) | 8.45 (43) | 1.18 (6) | 0.39 (2) | 0.39 (2) |
| BRCA | JNSERM 2016 | 213 | 25.35 | 2.82 (6) | 18.78 (40) | 3.29 (7) | 0.47 (1) | - |
| Lung Squ. | TCGA | 178 | 25.28 | 8.99 (16) | 11.24 (20) | 3.93 (7) | 1.12 (2) | - |
| Stomach/Oesophageal | TCGA | 265 | 24.53 | 7.17 (19) | 13.96 (37) | 1.51 (4) | 1.89 (5) | - |
| Stomach | TCGA | 393 | 23.92 | 11.96 (47) | 8.4 (33) | 2.29 (9) | 1.27 (5) | - |
| Breast | TCGA 2015 | 816 | 23.65 | 2.33 (19) | 19 (155) | 1.96 (16) | 0.37 (3) | - |
| Breast | TCGA | 963 | 23.47 | 2.08 (20) | 17.86 (172) | 2.91 (28) | 0.62 (6) | - |
| Melanoma | Broad | 121 | 23.14 | 23.14 (28) | - | - | - | - |
| Stomach | TCGA pub | 287 | 23 | 11.85 (34) | 6.62 (19) | 2.79 (8) | 1.74 (5) | - |
| Prostate | FHCRC, 2016 | 136 | 22.79 | 7.35 (10) | 8.82 (12) | 3.68 (5) | 2.94 (4) | - |
| Lung Squ. | TCGA PanCan | 469 | 21.75 | 10.02 (47) | 7.68 (36) | 2.13 (10) | 1.49 (7) | 0.43 (2) |
| Ovarian | TCGA PanCan | 398 | 21.36 | 4.02 (16) | 16.58 (66) | 0.5 (2) | 0.25 (1) | - |
| Oesophagus | TCGA PanCan | 182 | 20.88 | 6.59 (12) | 10.44 (19) | 1.65 (3) | 1.1 (2) | 1.1 (2) |
| Lung adeno. | TCGA pub | 230 | 20.87 | 6.52 (15) | 11.74 (27) | 0.87 (2) | 1.74 (4) | - |
| Uterine | TCGA pub | 240 | 20.83 | 9.58 (23) | 8.75 (21) | 1.67 (4) | 0.83 (2) | - |
| CCLE | Novartis/Broad2012 | 881 | 20.77 | - | 13.85 (122) | 6.92 (61) | - | - |
| Liver | TCGA | 366 | 20.49 | 4.37 (16) | 15.57 (57) | 0.55 (2) | - | - |
| Lung adeno. | TCGA | 230 | 20.43 | 6.52 (15) | 11.74 (27) | 0.87 (2) | 1.3 (3) | - |
| Sarcoma | TCGA | 243 | 20.16 | 2.88 (7) | 11.52 (28) | 5.35 (13) | 0.41 (1) | - |

**Supplementary Table S7**. Percentages of alterations in *PROM1, PROM2, GRP, ZNF157, FRZB, CLDN10, PIP5K1B, CDHR1, CX3CL1,* and *PDZD2* in various cancers (cBioPortal web).

| **Cancer** | **Data source** | ***PROM1*** | ***PROM2*** | ***GRP*** | ***ZNF157*** | ***FRZB*** | ***CLDN10*** | ***PIP5K1B*** | ***CDHR1*** | ***CX3CL1*** | ***PDZD2*** |
| --- | --- | --- | --- | --- | --- | --- | --- | --- | --- | --- | --- |
| NEPC | Trento/Cornell/Broad 2016 | 10% | 16% | 10% | 39% | 12% | 16% | 25% | 18% | 8% | 26% |
| Melanoma | TCGA PanCan Atlas | 7% | 9% | 4% | 9% | 4% | 8% | 11% | 6% | 7% | 27% |
| Lung Squ. | TCGA | 5% | 7% | 4% | 7% | 6% | 7% | 5% | 6% | 6% | 26% |
| Prostate | FHCRC, 2016 | 11% | 19% | 6% | 31% | 11% | 22% | 11% | 11% | 7% | 26% |
| Stomach | TCGA pub | 6% | 8% | 6% | 9% | 7% | 5% | 7% | 7% | 7% | 23% |
| Melanoma | TCGA | 4% | 10% | 5% | 6% | 4% | 6% | 9% | 5% | 6% | 19% |
| Pancreas | UTSW | 3% | 6% | 22% | 0% | 3% | 2% | 4% | 1% | 90% | 3% |
| Oesophagus | TCGA | 7% | 11% | 14% | 11% | 4% | 5% | 6% | 6% | 6% | 19% |
| Lung Squ. | TCGA PanCan Atlas | 5% | 7% | 3% | 7% | 6% | 7% | 6% | 7% | 6% | 26% |
| Ovarian | TCGA | 6% | 5% | 7% | 10% | 6% | 6% | 4% | 2% | 2% | 12% |
| CCLE | Novartis/Broad2012 | 12% | 8% | 12% | 0% | 6% | 14% | 10% | 1% | 9% | 17% |
| Stomach | TCGA | 6% | 6% | 5% | 8% | 6% | 5% | 8% | 5% | 5% | 15% |
| Bladder | TCGA, 2014 | 3% | 5% | 7% | 9% | 10% | 8% | 6% | 5% | 11% | 19% |
| Stomach/Oesophageal | TCGA | 6% | 6% | 5% | 8% | 6% | 5% | 8% | 5% | 5% | 15% |
| Bladder | TCGA | 4% | 5% | 10% | 5% | 4% | 6% | 5% | 5% | 5% | 14% |
| Uterine | TCGA PanCan | 5% | 3% | 3% | 6% | 4% | 3% | 3% | 4% | 3% | 9% |
| NSCLC | TCGA, 2016 | 2% | 2% | 2% | 3% | 3% | 2% | 2% | 4% | 1% | 14% |
| Sarcoma | TCGA | 8% | 7% | 5% | 13% | 3% | 6% | 7% | 3% | 3% | 10% |
| Lung Squ. | TCGA pub | 5% | 7% | 4% | 7% | 6% | 7% | 5% | 6% | 6% | 26% |
| Oesophagus | TCGA PanCan | 6% | 8% | 10% | 12% | 4% | 5% | 6% | 6% | 5% | 18% |
| Stomach | TCGA PanCan | 4% | 7% | 4% | 8% | 7% | 4% | 8% | 5% | 5% | 16% |
| BLCA | TCGA, 2017 | 5% | 6% | 11% | 6% | 4% | 6% | 7% | 7% | 5% | 16% |
| Lung adeno. | TCGA | 7% | 7% | 5% | 6% | 5% | 7% | 4% | 3% | 7% | 17% |
| Ovarian | TCGA PanCan | 6% | 7% | 9% | 16% | 8% | 6% | 4% | 4% | 3% | 13% |
| Lung adeno. | TCGA PanCan | 8% | 8% | 5% | 6% | 7% | 8% | 5% | 5% | 6% | 17% |
| The MBC project | WAGLE, 2017 | 4% | 4% | 5% | 0% | 0% | 4% | 3% | 8% | 1% | 6% |
| Lung adeno. | TCGA pub | 10% | 7% | 5% | 5% | 10% | 7% | 3% | 5% | 9% | 19% |
| Melanoma | Broad | 5% | 7% | 1% | 6% | 0% | 2% | 5% | 4% | 3% | 8% |
| Bladder | TCGA PanCan | 5% | 6% | 11% | 6% | 4% | 5% | 6% | 5% | 6% | 16% |
| Lung adeno. | Broad | 2% | 2% | 5% | 2% | 2% | 2% | 1% | 4% | 1% | 12% |

**Supplementary Table S8.** Percentages of alterations in *PROM2, PROM1, EHF, FAM110C, SGPP2, ADGRG1, CWH43, PRRG2, TSPAN1,* and *ELF3* in various cancers (cBioPortal web).

| **Cancer** | **Data source** | ***PROM2*** | ***PROM1*** | ***EHF*** | ***FAM110C*** | ***SGPP2*** | ***ADGRG1*** | ***CWH43*** | ***PRRG2*** | ***TSPAN1*** | ***ELF3*** |
| --- | --- | --- | --- | --- | --- | --- | --- | --- | --- | --- | --- |
| NEPC | Trento/Cornell/Broad 2016 | 16.0% | 10.0% | 16.0% | 22.0% | 13.0% | 6.0% | 16.0% | 21.0% | 16.0% | 32.0% |
| The MBC project | WAGLE, 2017 | 4.0% | 4.0% | 12.0% | 5.0% | 1.3% | 2.6% | 0.0% | 12.0% | 12.0% | 27.0% |
| BLCA | TCGA, 2017 | 2.7% | 2.0% | 5.0% | 5.0% | 3.0% | 2.5% | 1.5% | 2.5% | 3.0% | 13.0% |
| Bladder | TCGA PanCan Atlas | 6.0% | 5.0% | 7.0% | 6.0% | 6.0% | 6.0% | 8.0% | 8.0% | 8.0% | 18.0% |
| Melanoma | TCGA PanCan Atlas | 9.0% | 7.0% | 3.0% | 5.0% | 3.0% | 6.0% | 15.0% | 2.5% | 2.8% | 7.0% |
| Pancreas | UTSW | 6.0% | 2.8% | 4.0% | 1.8% | 2.8% | 1.8% | 1.8% | 15.0% | 6.0% | 9.0% |
| Bladder | TCGA | 5.0% | 4.0% | 7.0% | 7.0% | 6.0% | 5.0% | 7.0% | 7.0% | 8.0% | 9.0% |
| Breast | METABRIC | 4.0% | 4.0% | 5.0% | 5.0% | 3.0% | 0.0% | 4.0% | 6.0% | 3.0% | 23.0% |
| Bladder | TCGA, 2014 | 5.0% | 3.0% | 9.0% | 6.0% | 8.0% | 8.0% | 11.0% | 9.0% | 6.0% | 13.0% |
| Melanoma | TCGA | 10.0% | 4.0% | 4.0% | 9.0% | 4.0% | 6.0% | 18.0% | 5.0% | 2.8% | 7.0% |
| Ovarian | TCGA | 5.0% | 6.0% | 8.0% | 7.0% | 4.0% | 3.0% | 2.5% | 3.0% | 11.0% | 9.0% |
| Oesophagus | TCGA | 11.0% | 7.0% | 14.0% | 5.0% | 4.0% | 6.0% | 11.0% | 8.0% | 9.0% | 8.0% |
| Uterine | TCGA PanCan | 8.0% | 16.0% | 8.0% | 12.0% | 8.0% | 9.0% | 11.0% | 11.0% | 11.0% | 14.0% |
| BRCA | JNSERM 2016 | 1.4% | 1.4% | 8.0% | 2.3% | 0.9% | 2.3% | 2.3% | 2.3% | 0.9% | 8.0% |
| Lung Squ. | TCGA | 2.6% | 3.0% | 2.2% | 1.4% | 1.2% | 2.2% | 6.0% | 2.2% | 2.2% | 2.2% |
| Stomach/Oesophageal | TCGA | 1.9% | 1.9% | 8.0% | 2.6% | 1.1% | 2.3% | 4.0% | 2.3% | 1.1% | 5.0% |
| Stomach | TCGA | 3.0% | 4.0% | 6.0% | 2.0% | 1.5% | 4.0% | 2.5% | 1.3% | 0.8% | 5.0% |
| Breast | TCGA 2015 | 1.0% | 1.3% | 3.0% | 1.6% | 1.0% | 1.5% | 2.0% | 2.0% | 1.3% | 12.0% |
| Breast | TCGA | 0.9% | 1.9% | 3.0% | 1.8% | 0.9% | 2.3% | 1.9% | 1.9% | 1.5% | 11.0% |
| Melanoma | Broad | 7.0% | 5.0% | 1.7% | 0.8% | 0.8% | 2.5% | 11.0% | 0.0% | 1.7% | 1.7% |
| Stomach | TCGA pub | 3.0% | 3.0% | 5.0% | 1.7% | 1.4% | 3.0% | 2.8% | 1.0% | 1.0% | 6.0% |
| Prostate | FHCRC, 2016 | 4.0% | 4.0% | 6.0% | 7.0% | 9.0% | 4.0% | 4.0% | 7.0% | 6.0% | 15.0% |
| Lung Squ. | TCGA PanCan | 3.0% | 3.0% | 1.7% | 1.1% | 2.1% | 0.6% | 9.0% | 0.6% | 1.1% | 2.3% |
| Ovarian | TCGA PanCan | 2.5% | 2.5% | 3.0% | 2.5% | 0.8% | 1.3% | 2.3% | 1.0% | 7.0% | 4.0% |
| Oesophagus | TCGA PanCan | 1.6% | 0.5% | 8.0% | 0.5% | 2.7% | 2.2% | 5.0% | 1.6% | 0.5% | 2.2% |
| Lung adeno. | TCGA pub | 1.7% | 1.3% | 3.0% | 0.4% | 0.4% | 1.7% | 2.6% | 0.9% | 0.9% | 10.0% |
| Uterine | TCGA pub | 2.5% | 5.0% | 4.0% | 2.5% | 2.1% | 2.9% | 4.0% | 0.8% | 1.3% | 5.0% |
| CCLE | Novartis/Broad 2012 | 8.0% | 12.0% | 11.0% | 7.0% | 7.0% | 4.0% | 4.0% | 9.0% | 5.0% | 10.0% |
| Liver | TCGA | 1.1% | 3.0% | 0.8% | 2.5% | 0.8% | 1.1% | 1.4% | 0.8% | 0.5% | 11.0% |
| Lung adeno. | TCGA | 1.7% | 1.3% | 3.0% | 0.4% | 0.4% | 1.7% | 2.6% | 0.9% | 0.9% | 9.0% |
| Sarcoma | TCGA | 2.1% | 2.9% | 2.5% | 2.9% | 2.9% | 0.4% | 5.0% | 0.4% | 2.1% | 1.6% |

**Supplementary Figures**


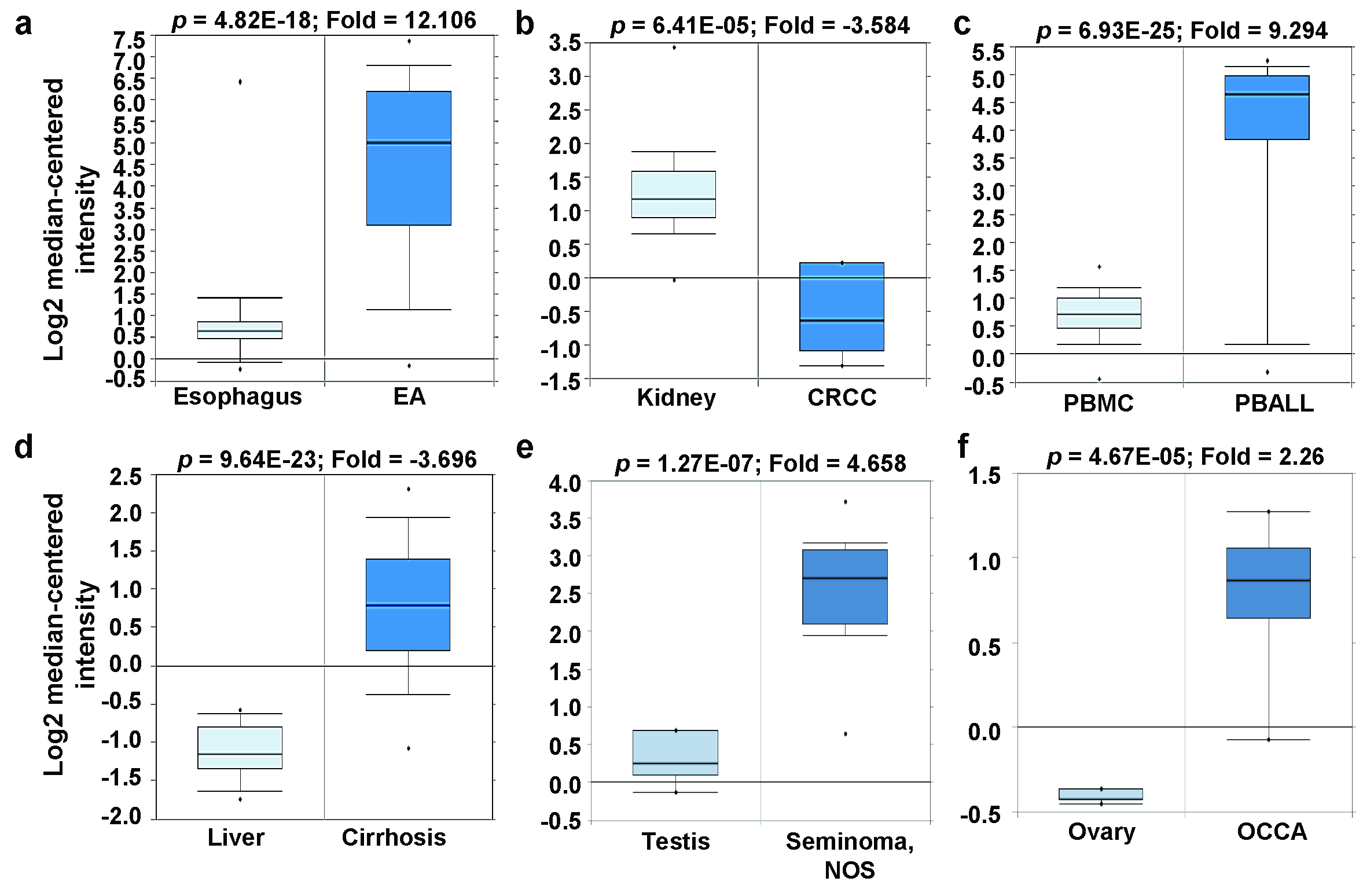


**Supplementary Figure S1.** *PROM1* expression analysis in different cancer types (Oncomine database). Box plot comparing specific *PROM1* expression in normal (left plot) and cancer tissue (right plot) was derived from the Oncomine database. The fold change of *PROM1* in various cancer types was identified from our analyses in Supplementary Table 1. The analysis was shown in EA relative to normal oesophagus **(a)**, CRCC relative to normal kidney **(b)**, PBALL relative to PBMC (**c**), cirrhosis relative to normal liver (**d**), seminoma, NOS relative to normal testis (**E**), and OCCA relative to normal ovary (**f**). Abbreviations: EA-Oesophageal adenocarcinoma; CRCC-Chromophobe renal cell carcinoma; PBMC-Peripheral blood mononuclear cell; PBALL-Pro-B acute lymphoblastic leukaemia; OCCA-Ovarian clear cell adenocarcinoma.


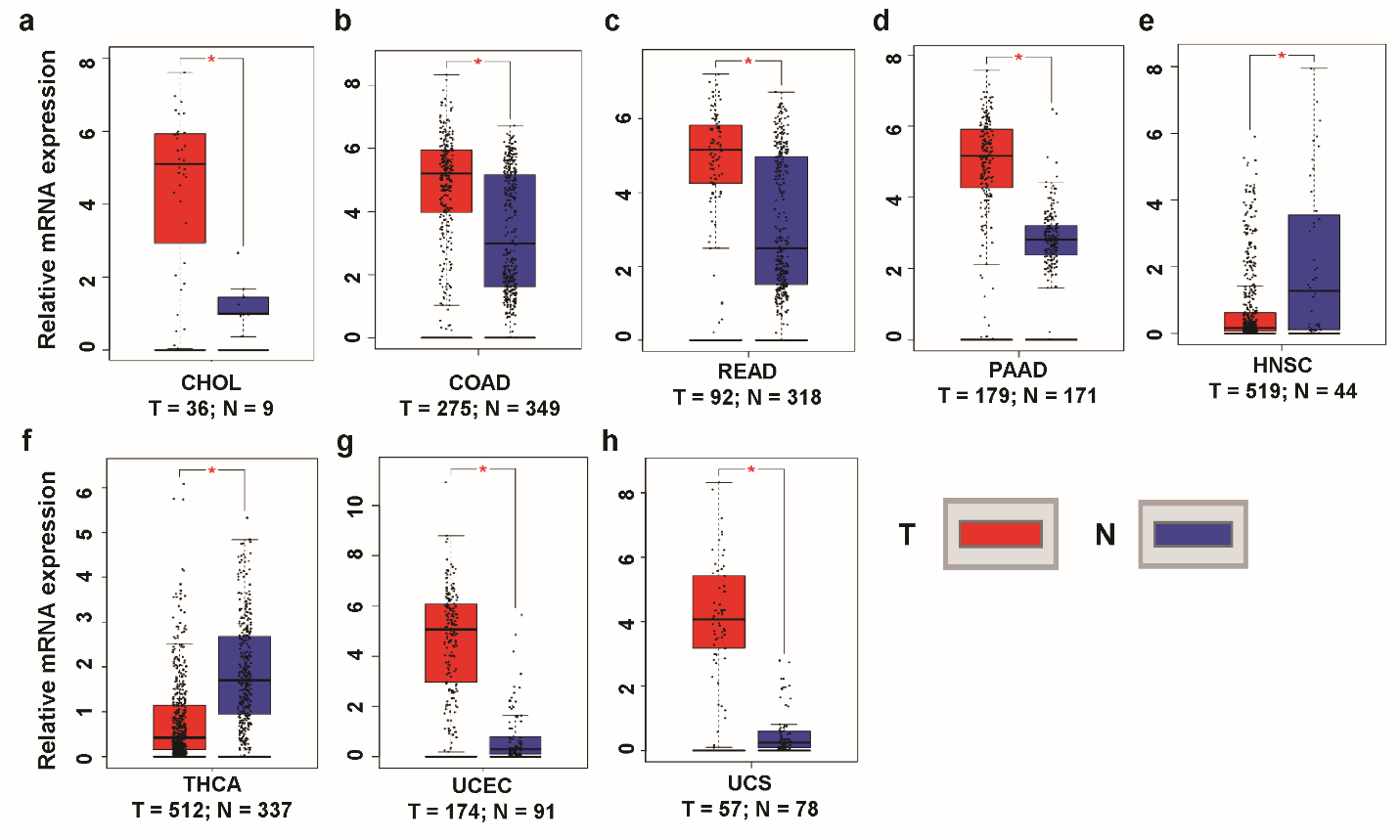


**Supplementary Figure S2. PROM1 expression analysis in different cancer types (TCGA database).** The box plot comparing specific PROM1 expression in cancer (left plot) and normal tissue (right plot) was derived from GEPIA database. The analysis was shown in CHOL relative to normal cholangio **(a)**, in COAD relative to normal colon **(b)**, in READ relative to normal rectum (**c**), in PAAD relative to normal Pancreas (**d**), HNSC relative to normal head and neck (**e**), in THCA relative to normal thyroid (**f**), in UCEC relative to normal uterine corpus endometrial  **(g)**, and in UCS relative to normal uterine (**h**). (Abbreviations: CHOL-Cholangio carcinoma; COAD-Colon adenocarcinoma; PAAD-Pancreatic adenocarcinoma; READ-Rectum adenocarcinoma; HNSC-Head and Neck squamous cell carcinoma; THCA-Thyroid carcinoma; UCEC-Uterine Corpus Endometrial Carcinoma; UCS-Uterine Carcinosarcoma).


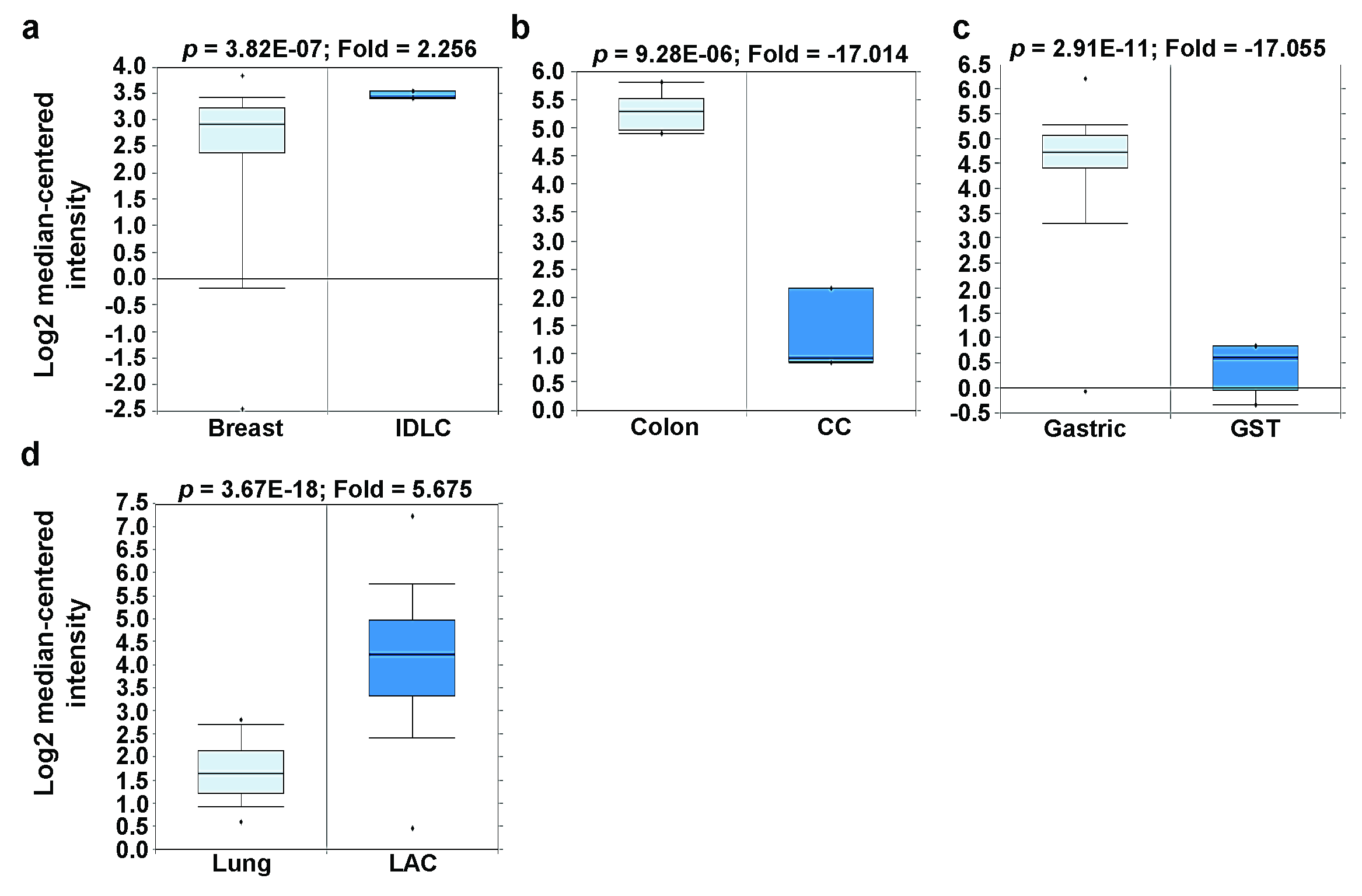


**Supplementary Figure S3.** *PROM2* expression analysis in different cancer types (Oncomine database). Box plot comparing specific *PROM2* expression in normal (left plot) and cancer tissue (right plot) was derived from Oncomine database. Fold change of *PROM2* in various cancer types was identified from our analyses in Supplementary Table 2. The analysis was shown in IDLC relative to normal breast (**a**), CC relative to normal colon **(b)**, GST relative to normal stomach (**c**), and LAC relative to normal lung (**d**). Abbreviations: IDLC-Invasive ductal and lobular carcinoma; CC-Colon carcinoma; GST-Gastrointestinal stromal tumour; LAC-Lung adenocarcinoma.


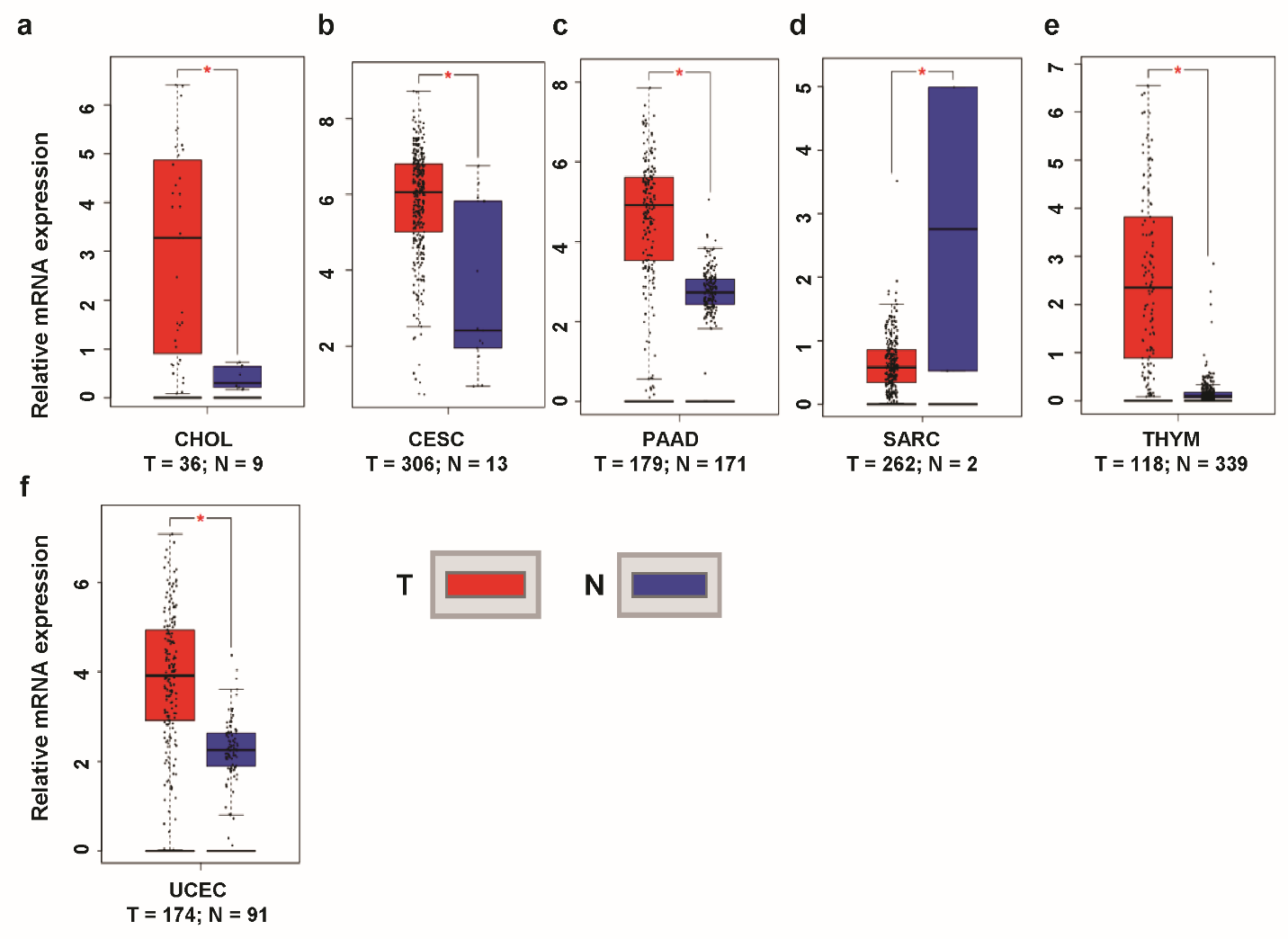


**Supplementary Figure S4. PROM2 expression analysis in different cancer types (TCGA database).** The box plot comparing specific PROM2 expression in cancer (left plot) and normal tissue (right plot) was derived from GEPIA database. The analysis was shown in CHOL relative to normal cholangio **(a)**, in CESC relative to normal cervix **(b)**, in PAAD relative to normal Pancreas (**c**), SARC relative to normal tissue (**d**), in THYM relative to normal thymoma (**e**), and in UCEC relative to normal uterine corpus endometrial (**f**). (Abbreviations: CHOL-Cholangio carcinoma; CESC-Cervical squamous cell carcinoma and endocervical adenocarcinoma; PAAD-Pancreatic adenocarcinoma; SARC-Sarcoma; THYM-Thymoma; UCEC-Uterine Corpus Endometrial Carcinoma).


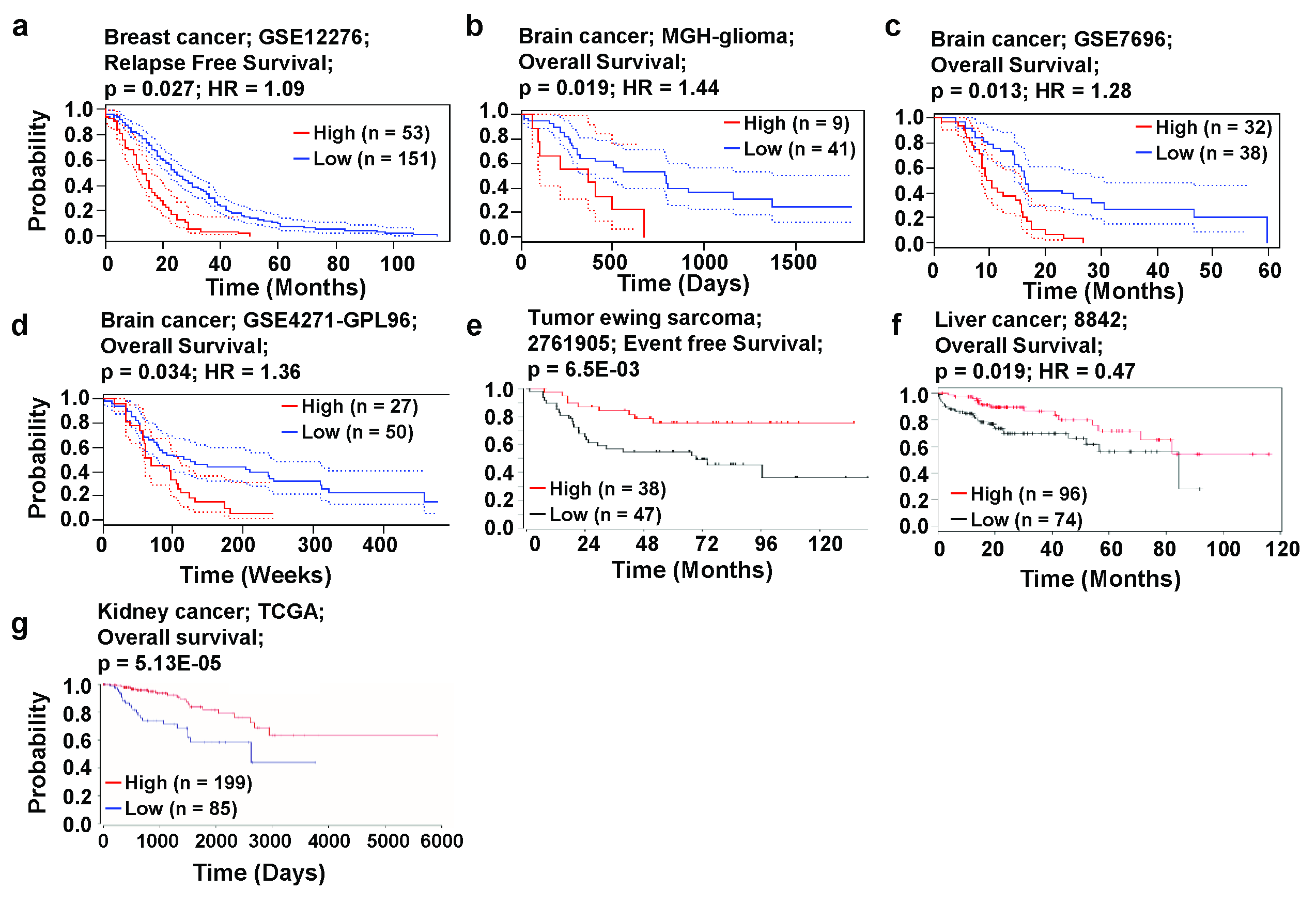


**Supplementary Figure S5.** Correlation of *PROM1* expression with breast, brain, sarcoma, kidney, and liver cancer prognosis (PrognoScan, R2, Kaplan–Meier plotter, and OncoLnc database). Survival curve comparing patients with high (red) and low (blue) expression of *PROM1* was plotted using data from PrognoScan database regarding breast cancer (**a**) and brain cancer (**b-d**). Survival curve comparing patients with high (red) and low (black) expression of *PROM1* was plotted using data from the R2 platform regarding Ewing sarcoma (**e**). Survival curve comparing patients with high (red) and low (black) *PROM1* expression was plotted using data from Kaplan–Meier plotter regarding liver cancer (**f**). Survival curve comparing patients with high (red) and low (blue) *PROM1* expression was plotted using data from the OncoLnc database regarding kidney cancer. Threshold of cox *p*-value < 0.05.


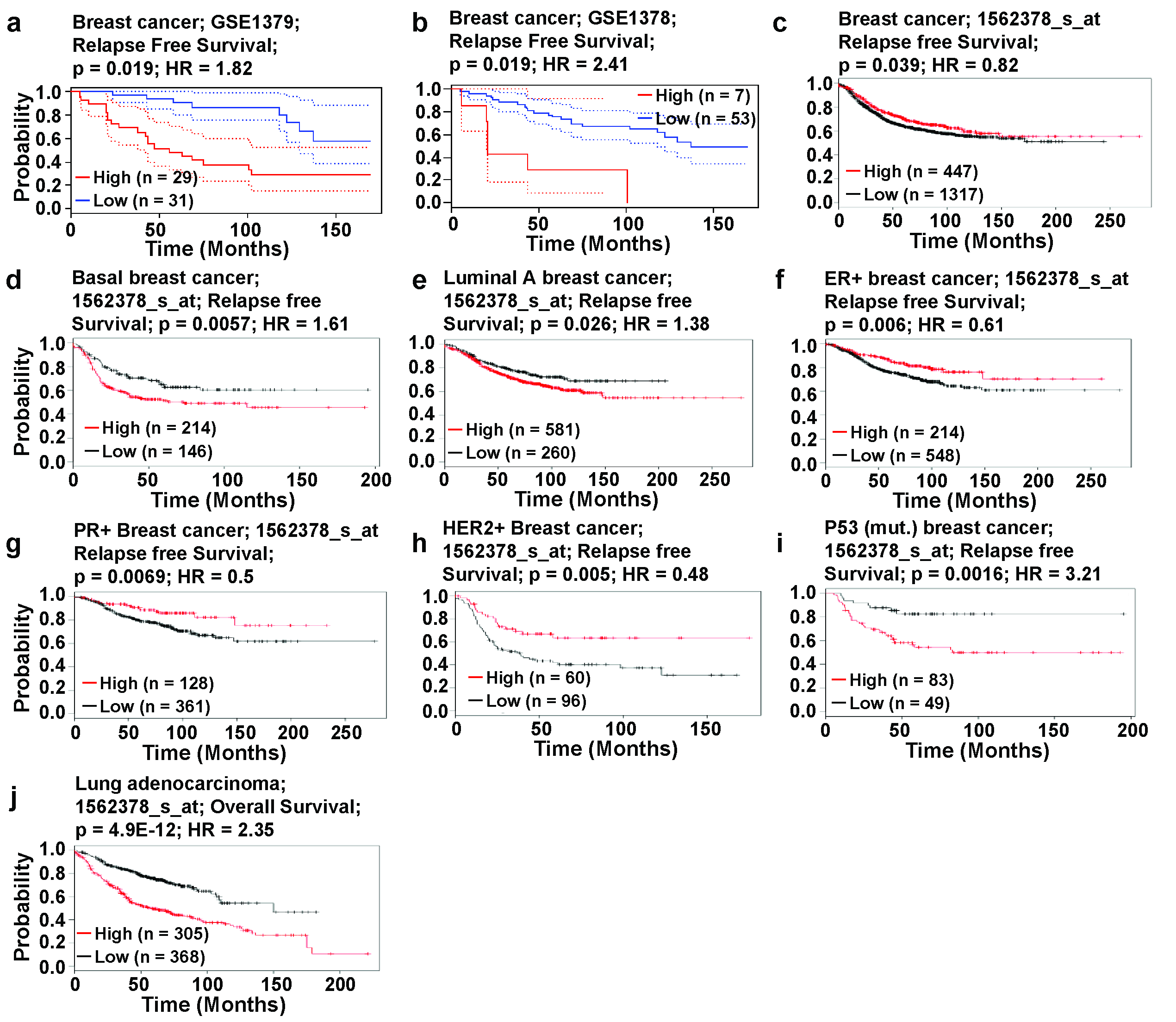


**Supplementary Figure S6.** Correlation of *PROM2* expression with breast cancer prognosis (PrognoScan and Kaplan–Meier plotter database). Survival curve comparing patients with high (red) and low (blue) *PROM1* expression was plotted using data from PrognoScan database regarding breast cancer (**a and b**). Survival curve comparing patients with high (red) and low (black) *PROM1* expression was plotted using data from Kaplan–Meier plotter database regarding various breast cancer subtypes (**c-i**). Survival curve comparing patients with high (red) and low (black) *PROM1* expression was plotted using data from Kaplan–Meier plotter database regarding lung adenocarcinoma (**j**). Threshold of Cox *p*-value < 0.05.


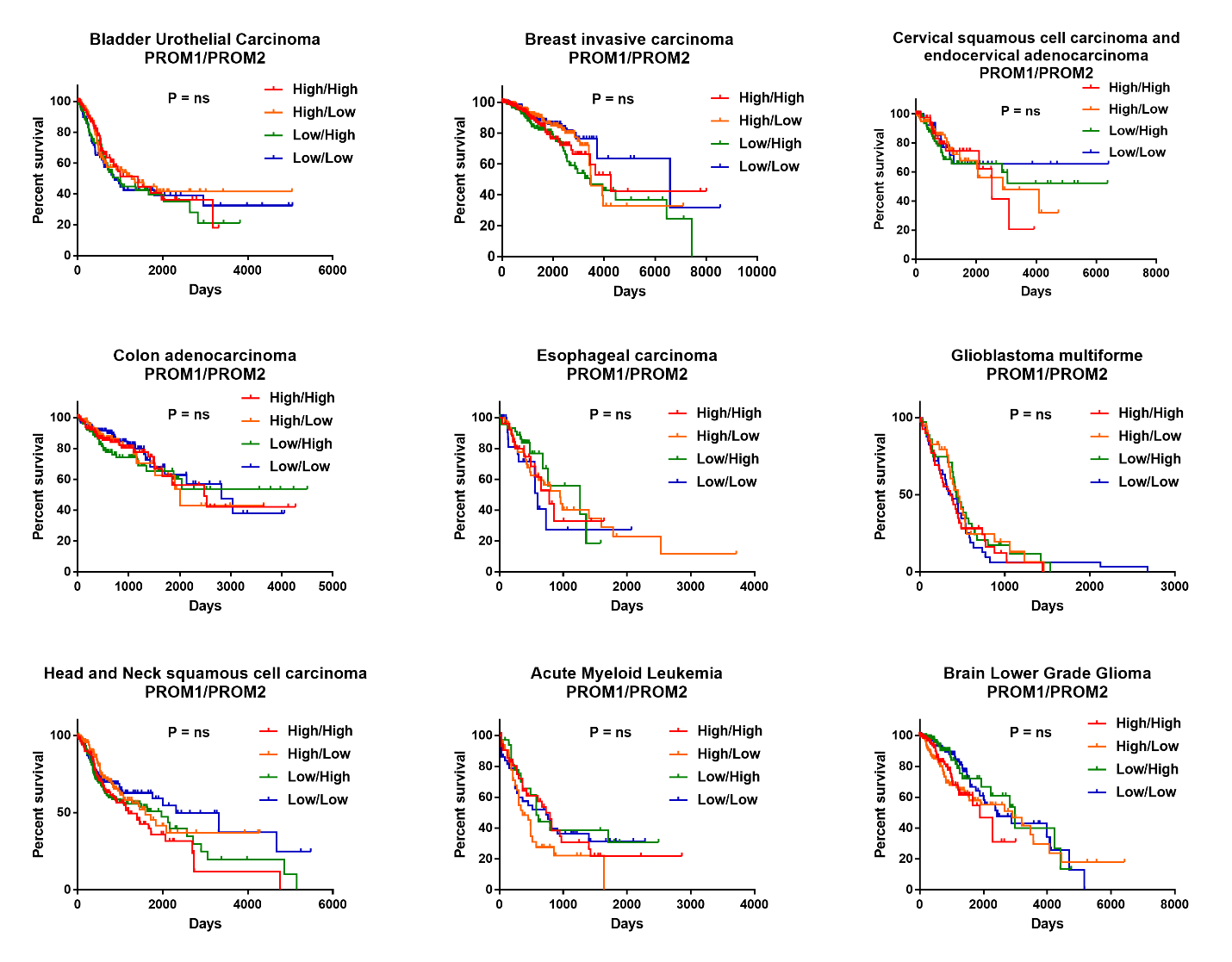


**Supplementary Figure S7.** Expression co-occurrence of *PROM1* and *PROM2* with respect to the clinical prognosis of cancer patients. Multivariate survival curves comparing clinical prognosis of patients with high/high (red), high/low (orange), low/high (green), and low/low (blue) expression co-occurrence of *PROM1/PROM2* in BLCA, BRCA, CESC, COAD, ESCA, GBM, HNSC, LAML, LIHC, LUAD, LUSC, OV, PAAD, READ, SARC, STAD, UCEC, and BLGG. The clinical outcome data were retrieved from the TCGA database using OncoLnc web. Information indicating statistical non-significance represents *p* = ns in the graph. Abbreviations: BLCA-Bladder urothelial carcinoma; BRCA-Breast invasive carcinoma; CESC-Cervical squamous cell carcinoma and endocervical adenocarcinoma; COAD-Colon adenocarcinoma; ESCA-Oesophageal carcinoma; GBM-Glioblastoma multiforme; HNSC-Head and neck squamous cell carcinoma; LAML-Acute myeloid leukaemia; BLGG-Brain lower grade glioma; LIHC-Liver hepatocellular carcinoma; LUAD-Lung adenocarcinoma; LUSC-Lung squamous cell carcinoma; OV-Ovarian serous cystadenocarcinoma; PAAD-Pancreatic adenocarcinoma; READ-Rectum adenocarcinoma; SARC-Sarcoma; STAD-Stomach adenocarcinoma; UCEC-Uterine corpus endometrial carcinoma.


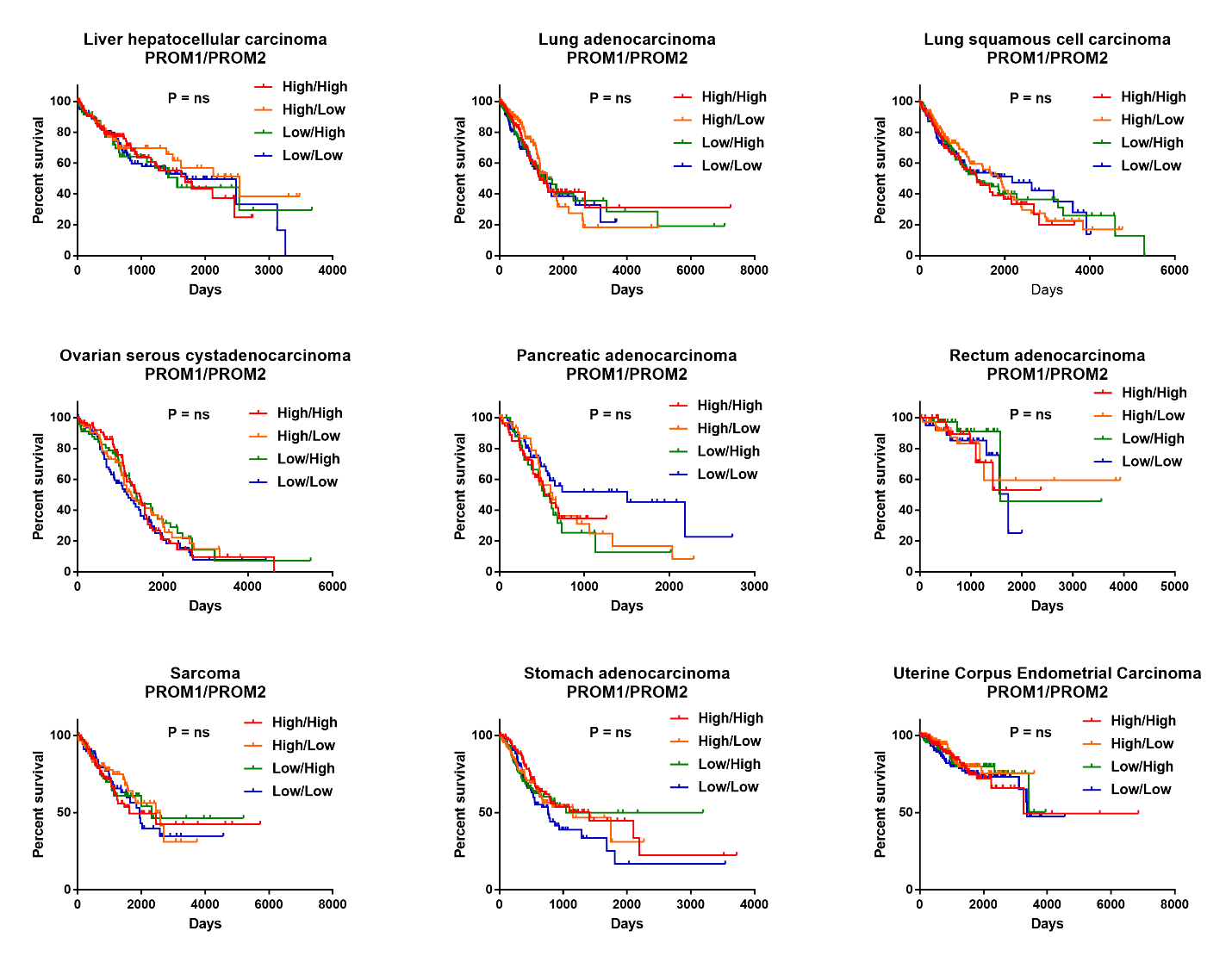


**Supplementary Figure S7.** (Continued).


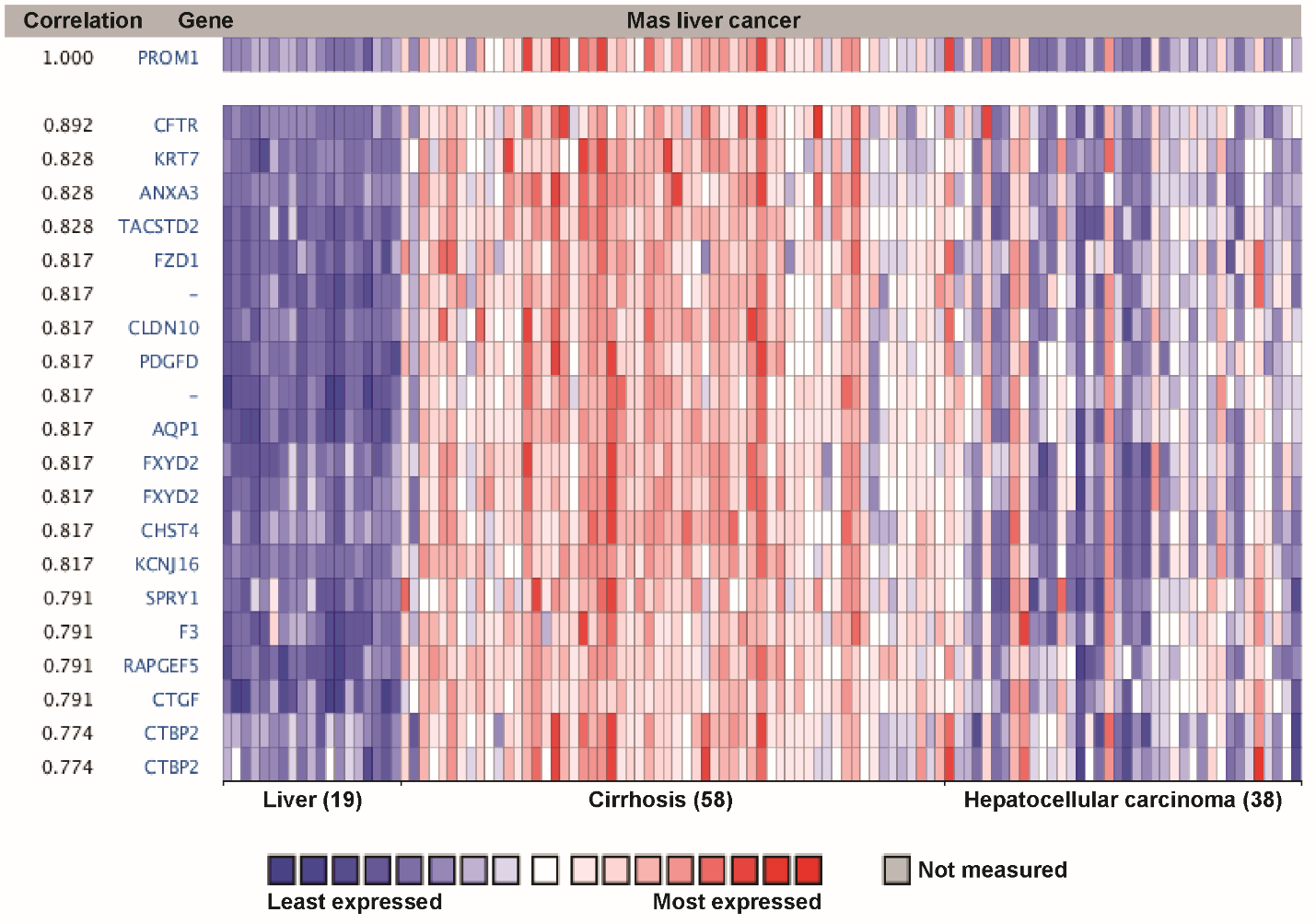


**Supplementary Figure S8.** Co-expression profile of *PROM1* in hepatocellular carcinoma. *PROM1* is co-expressed with the indicated genes across a panel of 19 hepatocellular carcinoma, 58 cirrhosis, and 19 normal liver samples. Bar lengths represent the significance and negative logarithm of enrichment *p*-value.


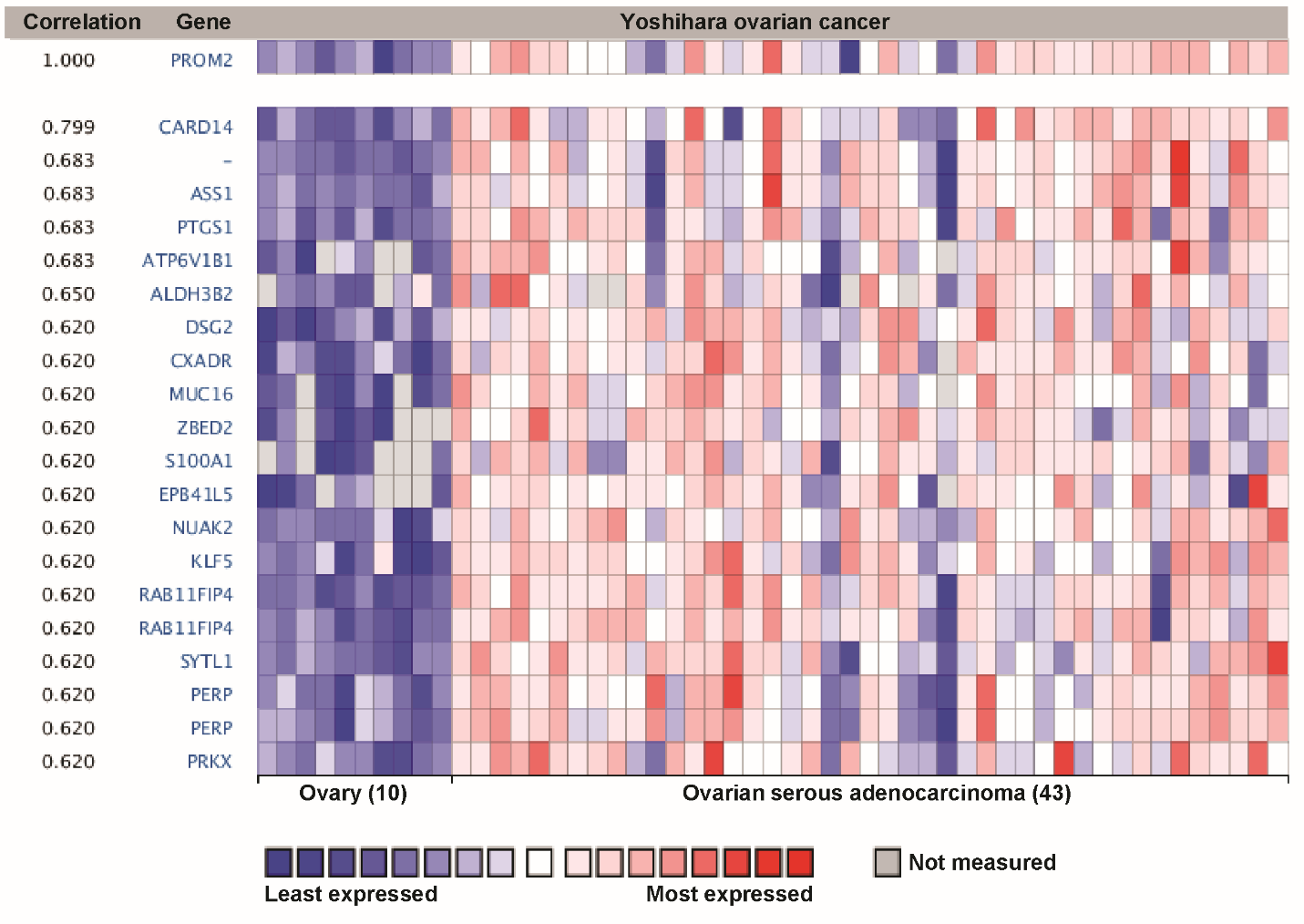


**Supplementary Figure S9.** Co-expression profile of *PROM2* in ovarian serous adenocarcinoma. *PROM2* is co-expressed with the indicated genes across a panel of 43 ovarian serous adenocarcinoma and 10 normal ovary samples. Bar lengths represent the significance and negative logarithm of enrichment *p*-value.


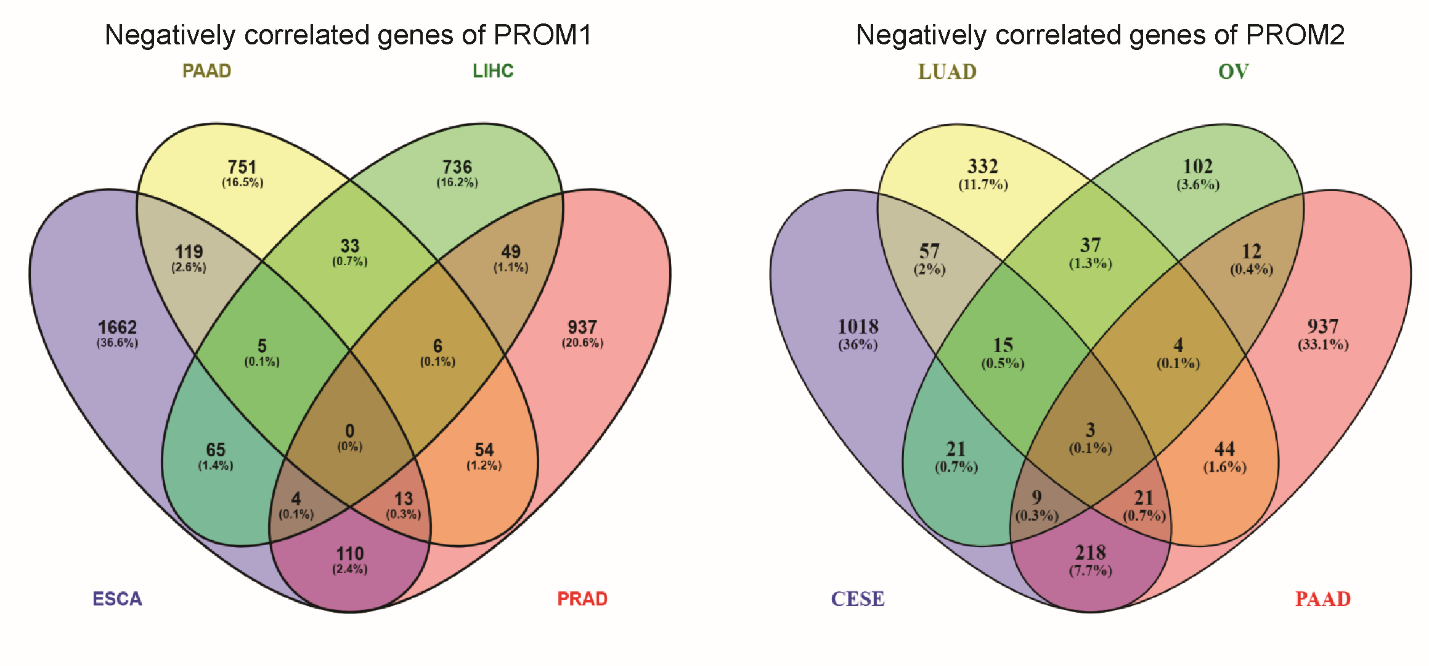


**Supplementary Figure S10.** Venn diagram of genes negatively correlated to *PROM1 and* *PROM2* showing coincident genes.


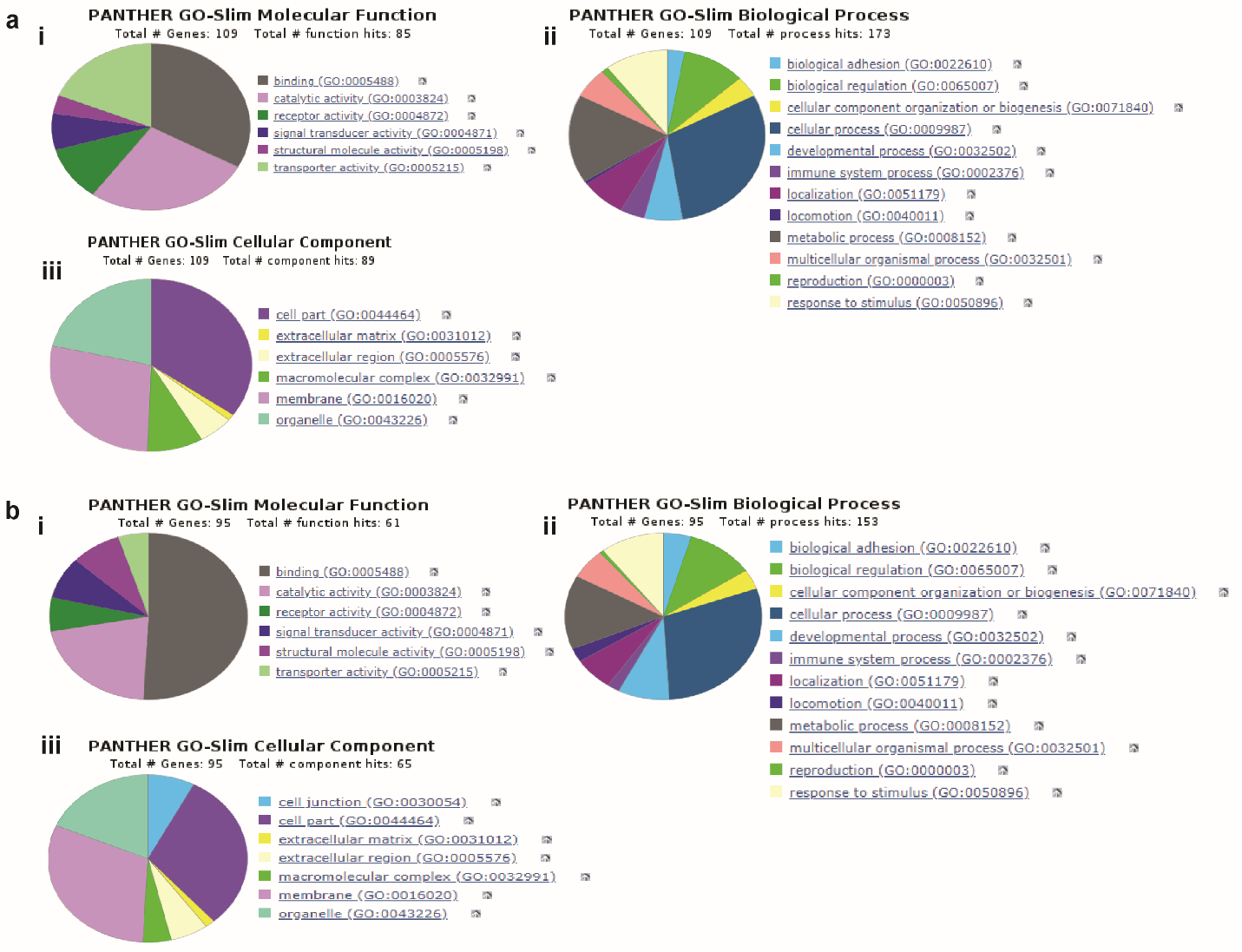


**Supplementary Figure S11.** Gene ontology (GO) analysis of positively correlated genes of *PROM1* and *PROM2* using PANTHER. **(A)** Gene ontology (GO) analysis of positively correlated genes of *PROM1* using PANTHER and subsequently classified based on their Molecular function (**i**), Biological Process (**ii**), and Cellular component (**iii**). (**B**) Gene ontology (GO) analysis of positively correlated genes of *PROM2* using PANTHER and subsequently classified based on their Molecular function (**i**), Biological Process (**ii**), and Cellular component (**iii**).

**References**

1. Lee, J.-S.; Leem, S.-H.; Lee, S.-Y.; Kim, S.-C.; Park, E.-S.; Kim, S.-B.; Kim, S.-K.; Kim, Y.-J.; Kim, W.-J.; Chu, I.-S. Expression signature of E2F1 and its associated genes predict superficial to invasive progression of bladder tumors. *J. Clin. Oncol.* **2010**, *28*, 2660-2667.

2. Murat, A.; Migliavacca, E.; Gorlia, T.; Lambiv, W.L.; Shay, T.; Hamou, M.-F.; De Tribolet, N.; Regli, L.; Wick, W.; Kouwenhoven, M.C. Stem cell–related “self-renewal” signature and high epidermal growth factor receptor expression associated with resistance to concomitant chemoradiotherapy in glioblastoma. *J. Clin. Oncol.* **2008**, *26*, 3015-3024.

3. Curtis, C.; Shah, S.P.; Chin, S.-F.; Turashvili, G.; Rueda, O.M.; Dunning, M.J.; Speed, D.; Lynch, A.G.; Samarajiwa, S.; Yuan, Y. The genomic and transcriptomic architecture of 2,000 breast tumours reveals novel subgroups. *Nature* **2012**, *486*, 346.

4. Kim, S.M.; Park, Y.-Y.; Park, E.S.; Cho, J.Y.; Izzo, J.G.; Zhang, D.; Kim, S.-B.; Lee, J.H.; Bhutani, M.S.; Swisher, S.G. Prognostic biomarkers for esophageal adenocarcinoma identified by analysis of tumor transcriptome. *PloS One* **2010**, *5*, e15074.

5. Hao, Y.; Triadafilopoulos, G.; Sahbaie, P.; Young, H.S.; Omary, M.B.; Lowe, A.W. Gene expression profiling reveals stromal genes expressed in common between Barrett’s esophagus and adenocarcinoma. *Gastroenterology* **2006**, *131*, 925-933.

6. Cutcliffe, C.; Kersey, D.; Huang, C.C.; Zeng, Y.; Walterhouse, D.; Perlman, E.J. Clear cell sarcoma of the kidney: up-regulation of neural markers with activation of the sonic hedgehog and Akt pathways. *Clin. Cancer Res.* **2005**, *11*, 7986-7994.

7. Beroukhim, R.; Brunet, J.P.; Di Napoli, A.; Mertz, K.D.; Seeley, A.; Pires, M.M.; Linhart, D.; Worrell, R.A.; Moch, H.; Rubin, M.A., et al. Patterns of gene expression and copy-number alterations in von-hippel lindau disease-associated and sporadic clear cell carcinoma of the kidney. *Cancer Res.* **2009**, *69*, 4674-4681.

8. Jones, J.; Otu, H.; Spentzos, D.; Kolia, S.; Inan, M.; Beecken, W.D.; Fellbaum, C.; Gu, X.; Joseph, M.; Pantuck, A.J. Gene signatures of progression and metastasis in renal cell cancer. *Clin. Cancer Res.* **2005**, *11*, 5730-5739.

9. Haferlach, T.; Kohlmann, A.; Wieczorek, L.; Basso, G.; Kronnie, G.T.; Bene, M.C.; De Vos, J.; Hernandez, J.M.; Hofmann, W.K.; Mills, K.I., et al. Clinical utility of microarray-based gene expression profiling in the diagnosis and subclassification of leukemia: report from the International Microarray Innovations in Leukemia Study Group. *J. Clin. Oncol.* **2010**, *28*, 2529-2537.

10. Wurmbach, E.; Chen, Y.b.; Khitrov, G.; Zhang, W.; Roayaie, S.; Schwartz, M.; Fiel, I.; Thung, S.; Mazzaferro, V.; Bruix, J. Genome‐wide molecular profiles of HCV‐induced dysplasia and hepatocellular carcinoma. *Hepatology* **2007**, *45*, 938-947.

11. Mas, V.R.; Maluf, D.G.; Archer, K.J.; Yanek, K.; Kong, X.; Kulik, L.; Freise, C.E.; Olthoff, K.M.; Ghobrial, R.M.; McIver, P. Genes involved in viral carcinogenesis and tumor initiation in hepatitis C virus-induced hepatocellular carcinoma. *Mol. Med.* **2009**, *15*, 85.

12. Chen, X.; Cheung, S.T.; So, S.; Fan, S.T.; Barry, C.; Higgins, J.; Lai, K.M.; Ji, J.; Dudoit, S.; Ng, I.O., et al. Gene expression patterns in human liver cancers. *Mol. Biol. Cell* **2002**, *13*, 1929-1939.

13. Talantov, D.; Mazumder, A.; Jack, X.Y.; Briggs, T.; Jiang, Y.; Backus, J.; Atkins, D.; Wang, Y. Novel genes associated with malignant melanoma but not benign melanocytic lesions. *Clin. Cancer Res.* **2005**, *11*, 7234-7242.

14. Skotheim, R.I.; Lind, G.E.; Monni, O.; Nesland, J.M.; Abeler, V.M.; Fosså, S.D.; Duale, N.; Brunborg, G.; Kallioniemi, O.; Andrews, P.W. Differentiation of human embryonal carcinomas in vitro and in vivo reveals expression profiles relevant to normal development. *Cancer Res.* **2005**, *65*, 5588-5598.

15. Korkola, J.E.; Houldsworth, J.; Chadalavada, R.S.; Olshen, A.B.; Dobrzynski, D.; Reuter, V.E.; Bosl, G.J.; Chaganti, R. Down-regulation of stem cell genes, including those in a 200-kb gene cluster at 12p13. 31, is associated with in vivo differentiation of human male germ cell tumors. *Cancer Res.* **2006**, *66*, 820-827.

16. Hendrix, N.D.; Wu, R.; Kuick, R.; Schwartz, D.R.; Fearon, E.R.; Cho, K.R. Fibroblast growth factor 9 has oncogenic activity and is a downstream target of Wnt signaling in ovarian endometrioid adenocarcinomas. *Cancer Res.* **2006**, *66*, 1354-1362.

17. Cho, J.Y.; Lim, J.Y.; Cheong, J.H.; Park, Y.-Y.; Yoon, S.-L.; Kim, S.M.; Kim, S.-B.; Kim, H.; Hong, S.W.; Park, Y.N. Gene expression signature–based prognostic risk score in gastric cancer. *Clin. Cancer Res.* **2011**.

18. Karnoub, A.E.; Dash, A.B.; Vo, A.P.; Sullivan, A.; Brooks, M.W.; Bell, G.W.; Richardson, A.L.; Polyak, K.; Tubo, R.; Weinberg, R.A. Mesenchymal stem cells within tumour stroma promote breast cancer metastasis. *Nature* **2007**, *449*, 557.

19. Skrzypczak, M.; Goryca, K.; Rubel, T.; Paziewska, A.; Mikula, M.; Jarosz, D.; Pachlewski, J.; Oledzki, J.; Ostrowsk, J. Modeling oncogenic signaling in colon tumors by multidirectional analyses of microarray data directed for maximization of analytical reliability. *PloS One* **2010**, *5*, e13091.

20. Kaiser, S.; Park, Y.-K.; Franklin, J.L.; Halberg, R.B.; Yu, M.; Jessen, W.J.; Freudenberg, J.; Chen, X.; Haigis, K.; Jegga, A.G. Transcriptional recapitulation and subversion of embryonic colon development by mouse colon tumor models and human colon cancer. *Genome Biol.* **2007**, *8*, R131.

21. Hong, Y.; Downey, T.; Eu, K.W.; Koh, P.K.; Cheah, P.Y. A ‘metastasis-prone’signature for early-stage mismatch-repair proficient sporadic colorectal cancer patients and its implications for possible therapeutics. *Clin. Exp. Metastasis* **2010**, *27*, 83-90.

22. Gaedcke, J.; Grade, M.; Jung, K.; Camps, J.; Jo, P.; Emons, G.; Gehoff, A.; Sax, U.; Schirmer, M.; Becker, H. Mutated KRAS results in overexpression of DUSP4, a MAP‐kinase phosphatase, and SMYD3, a histone methyltransferase, in rectal carcinomas. *Genes, Chromosomes Cancer* **2010**, *49*, 1024-1034.

23. Yusenko, M.V.; Kuiper, R.P.; Boethe, T.; Ljungberg, B.; van Kessel, A.G.; Kovacs, G. High-resolution DNA copy number and gene expression analyses distinguish chromophobe renal cell carcinomas and renal oncocytomas. *BMC Cancer* **2009**, *9*, 152.

24. Selamat, S.A.; Chung, B.S.; Girard, L.; Zhang, W.; Zhang, Y.; Campan, M.; Siegmund, K.D.; Koss, M.N.; Hagen, J.A.; Lam, W.L. Genome-scale analysis of DNA methylation in lung adenocarcinoma and integration with mRNA expression. *Genome Res.* **2012**.

25. Okayama, H.; Kohno, T.; Ishii, Y.; Shimada, Y.; Shiraishi, K.; Iwakawa, R.; Furuta, K.; Tsuta, K.; Shibata, T.; Yamamoto, S. Identification of Genes Up-regulated in ALK-positive and EGFR/KRAS/ALK-negative Lung Adenocarcinomas. *Cancer Res.* **2011**, canres. 1403.2011.

26. Garber, M.E.; Troyanskaya, O.G.; Schluens, K.; Petersen, S.; Thaesler, Z.; Pacyna-Gengelbach, M.; van de Rijn, M.; Rosen, G.D.; Perou, C.M.; Whyte, R.I., et al. Diversity of gene expression in adenocarcinoma of the lung. *Proc. Natl. Acad. Sci. U S A* **2001**, *98*, 13784-13789.

27. Hou, J.; Aerts, J.; Den Hamer, B.; Van Ijcken, W.; Den Bakker, M.; Riegman, P.; van der Leest, C.; van der Spek, P.; Foekens, J.A.; Hoogsteden, H.C. Gene expression-based classification of non-small cell lung carcinomas and survival prediction. *PloS One* **2010**, *5*, e10312.

28. Piccaluga, P.P.; Agostinelli, C.; Califano, A.; Rossi, M.; Basso, K.; Zupo, S.; Went, P.; Klein, U.; Zinzani, P.L.; Baccarani, M. Gene expression analysis of peripheral T cell lymphoma, unspecified, reveals distinct profiles and new potential therapeutic targets. *J. Clin. Invest.* **2007**, *117*, 823-834.

29. Zhan, F.; Barlogie, B.; Arzoumanian, V.; Huang, Y.; Williams, D.R.; Hollmig, K.; Pineda-Roman, M.; Tricot, G.; van Rhee, F.; Zangari, M. Gene-expression signature of benign monoclonal gammopathy evident in multiple myeloma is linked to good prognosis. *Blood* **2007**, *109*, 1692-1700.

30. Riker, A.I.; Enkemann, S.A.; Fodstad, O.; Liu, S.; Ren, S.; Morris, C.; Xi, Y.; Howell, P.; Metge, B.; Samant, R.S. The gene expression profiles of primary and metastatic melanoma yields a transition point of tumor progression and metastasis. *BMC Med. Genomics* **2008**, *1*, 13.

31. Lu, K.H.; Patterson, A.P.; Wang, L.; Marquez, R.T.; Atkinson, E.N.; Baggerly, K.A.; Ramoth, L.R.; Rosen, D.G.; Liu, J.; Hellstrom, I. Selection of potential markers for epithelial ovarian cancer with gene expression arrays and recursive descent partition analysis. *Clin. Cancer Res.* **2004**, *10*, 3291-3300.

32. Yoshihara, K.; Tajima, A.; Komata, D.; Yamamoto, T.; Kodama, S.; Fujiwara, H.; Suzuki, M.; Onishi, Y.; Hatae, M.; Sueyoshi, K., et al. Gene expression profiling of advanced-stage serous ovarian cancers distinguishes novel subclasses and implicates ZEB2 in tumor progression and prognosis. *Cancer Sci.* **2009**, *100*, 1421-1428.

33. Grasso, C.S.; Wu, Y.-M.; Robinson, D.R.; Cao, X.; Dhanasekaran, S.M.; Khan, A.P.; Quist, M.J.; Jing, X.; Lonigro, R.J.; Brenner, J.C., et al. The mutational landscape of lethal castration-resistant prostate cancer. *Nature* **2012**, *487*, 239.
